# 3D Camouflage in an Ornithischian Dinosaur

**DOI:** 10.1016/j.cub.2016.06.065

**Published:** 2016-09-26

**Authors:** Jakob Vinther, Robert Nicholls, Stephan Lautenschlager, Michael Pittman, Thomas G. Kaye, Emily Rayfield, Gerald Mayr, Innes C. Cuthill

**Affiliations:** 1 School of Biological Sciences, University of Bristol, Life Sciences Building, 24 Tyndall Avenue, Bristol BS8 1TQ, UK; 2 School of Earth Sciences, University of Bristol, Wills Memorial Building, Queens Road, Bristol BS8 1RJ, UK; 3 Palaeocreations, 35 Hopps Road, Kingswood, Bristol BS15 9QQ, UK; 4 Vertebrate Palaeontology Laboratory, Department of Earth Sciences, The University of Hong Kong, Pokfulam, Hong Kong; 5 Burke Museum of Natural History and Culture, 4331 Memorial Way Northeast, Seattle, WA 98195, USA; 6 Department of Ornithology, Senckenberg Research Institute and Natural History Museum, Senckenberganlage 25, 60325 Frankfurt, Germany

**Keywords:** defensive coloration, countershading, paleocolor, Jehol biota, Yixian Formation, paleoenvironment, behavioral ecology, taphonomy, soft-tissue preservation, Lagerstätte

## Abstract

Countershading was one of the first proposed mechanisms of camouflage [1, 2]. A dark dorsum and light ventrum counteract the gradient created by illumination from above, obliterating cues to 3D shape [3, 4, 5, 6]. Because the optimal countershading varies strongly with light environment [7, 8, 9], pigmentation patterns give clues to an animal’s habitat. Indeed, comparative evidence from ungulates [9] shows that interspecific variation in countershading matches predictions: in open habitats, where direct overhead sunshine dominates, a sharp dark-light color transition high up the body is evident; in closed habitats (e.g., under forest canopy), diffuse illumination dominates and a smoother dorsoventral gradation is found. We can apply this approach to extinct animals in which the preservation of fossil melanin allows reconstruction of coloration [10, 11, 12, 13, 14, 15]. Here we present a study of an exceptionally well-preserved specimen of *Psittacosaurus* sp. from the Chinese Jehol biota [16, 17]. This *Psittacosaurus* was countershaded [16] with a light underbelly and tail, whereas the chest was more pigmented. Other patterns resemble disruptive camouflage, whereas the chin and jugal bosses on the face appear dark. We projected the color patterns onto an anatomically accurate life-size model in order to assess their function experimentally. The patterns are compared to the predicted optimal countershading from the measured radiance patterns generated on an identical uniform gray model in direct versus diffuse illumination. These studies suggest that *Psittacosaurus* sp. inhabited a closed habitat such as a forest with a relatively dense canopy.

**Video Abstract:**

## Highlights

• Preserved pigments in the dinosaur *Psittacosaurus* suggest countershading camouflage
• We predicted the optimal countershading camouflage for different light environments
• The dinosaur’s patterns would have been cryptic in a forest, but not open, habitat
• We can also infer that dinosaur predators used shape-from-shading cues to detect prey

## Graphical Abstract

**Figure:**
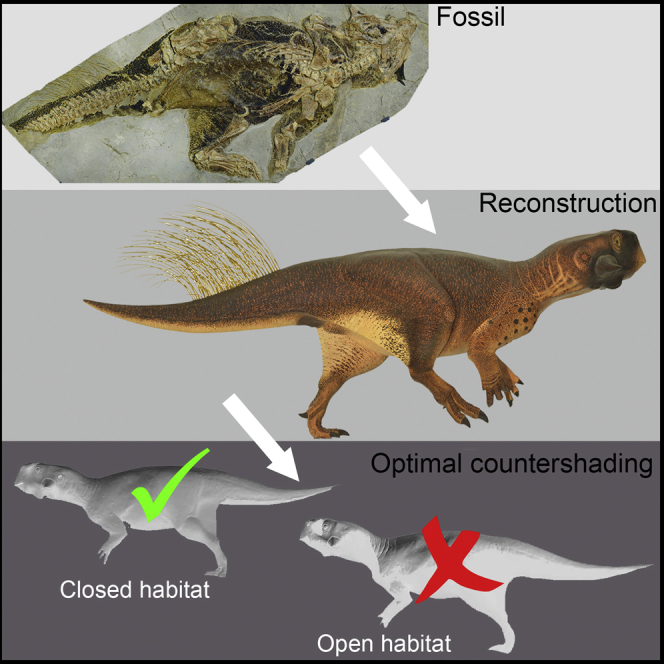

## Teaser

Countershading camouflage uses a dark-to-light gradient from back to belly to counter the light-to-dark gradient created by illumination. The body appears flatter and less conspicuous. Vinther et al. use 3D reconstruction and radiance modeling to show that the dinosaur *Psittacosaurus* was countershaded and cryptic in a forested environment.

## Results

The past years have witnessed an increased interest in the coloration of fossil animals. Studies of fossil melanosomes in particular have allowed reconstruction of the plumage patterns of theropod dinosaurs [12, 13, 15] and color in dinosaurs and other extinct vertebrates [14, 18]. Most of these studies focused on the coloration of integumental appendages, such as feathers, whereas the skin coloration of non-feathered dinosaurs remains little studied.

**Figure 1:**
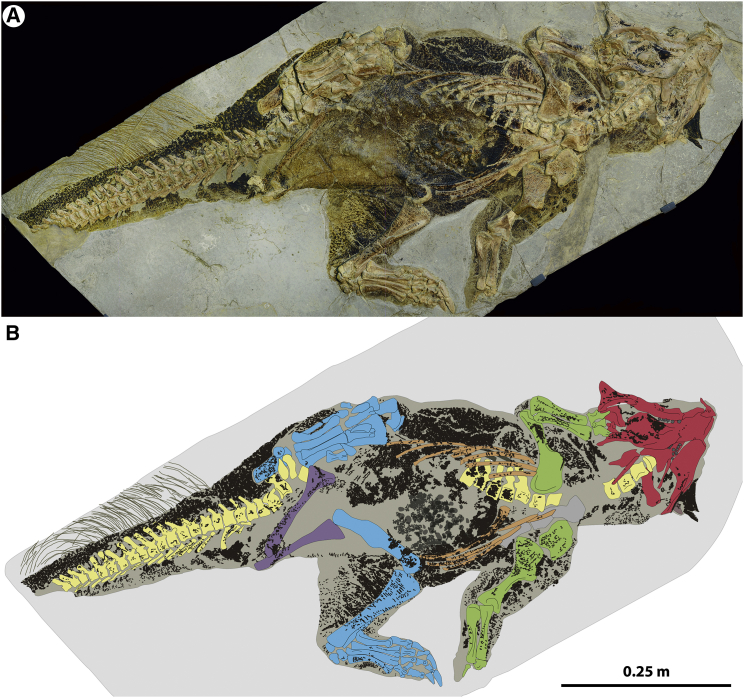
*Psittacosaurus sp*. SMF R 4970, Whole Specimen (A) Specimen photographed under crossed polarized light. (B) Interpretative drawing, showing the distribution of pigment patterns, skin, and bones. Green indicates forelimb bones, blue indicates hindlimb bones, purple indicates sacral elements, red indicates cranial elements, and buff yellow indicates vertebral column.

Here we study a ceratopsian ornithischian, *Psittacosaurus* sp. (SMF R 4970), from the Early Cretaceous Jehol Biota, with tail filaments and extremely well preserved skin preservation as a compressed film outlining the body, superimposed on the skeleton [17] (Figure 1). The specimen has had a tumultuous history [19, 20], but it has been on public display at the Senckenberg Museum in Frankfurt for the last 12 years. Most of the integument probably has been preserved as a calcium phosphate residue from the mineral salts embedded within the scales [21] to harden them. Calcium phosphate fluoresces strongly under laser illumination [22], allowing for detailed analysis of the scale-clad integument (Supplemental Experimental Procedures and Figures S2 and S3). The specimen preserves distinct color patterns suggesting countershading, which has been noted earlier [16]. As yet, however, no detailed description of the soft-tissue preservation of this exceptional fossil exists, nor has the proposed countershading been quantitatively examined and tested.

In the present study, the reconstructed color patterns were based on the distribution of organic residues, which were made discernible through crossed polarized light photography and laser-stimulated fluorescence (LSF) imaging (Supplemental Experimental Procedures). The color patterns visible in the fossil are derived from preserved melanin on the specimen evident as melanosome-shaped structures under the electron microscope (Figure S4). These have been projected onto a 3D model created using the best available volumetric evidence about its skeletal posture and musculature (Supplemental Experimental Procedures). This was performed blind to any data on the predicted optimal countershading for different lighting conditions. Taken together, exceptional fossil evidence of color patterns and the link between countershading and habitat in extant taxa present a novel opportunity to predict habitat occupation in extinct taxa in which coloration patterns are preserved.

### Integumental Taphonomy

Distinct scales are present on the specimen (Figures S3 and S4). The scales are generally of similar dimensions across the body but are larger over the distal end of the ischium, forming a likely callosity, and over the ankle. Finer scales are seen on the toe and finger pads. The inner thighs, legs, and lower part of the face were scale free, whereas scales across the abdomen and ventral tail are dense and generally rectangular in shape (Figures S4G and S4H). The integument varies in the presence of dark-colored organic material, which we interpret as melanin residues from original skin pigmentation, as has been noted earlier [16]. This assumption is also supported by the presence of ovoid impressions similar in shape to phaeomelanosomes (Figure S5), which would suggest a brown color, as suggested by canonical discriminant analysis [12] and consistent with observations made in two other psittacosaur specimens from the Jehol biota [23] (Figure S5). It is debated, however, how melanosome shape correlates to melanin chemistry outside of mammals and birds [23] (but see [18, 24] for an alternative stance).

Internal organs, such as the liver, may also contain melanin [24, 25]. We observe organic residues from within the body in SMF R 4970, but the spatial distribution clearly distinguishes this from integumentary melanin (Figure S6; see the Supplemental Experimental Procedures). We reject the notion that the dark, organic residues are derived from keratin due to their local distribution, which does not correlate to scale thickness, and wider spatial distribution (see the Supplemental Experimental Procedures). We also do not find decay likely to be responsible for the observed patterns across SMF R 4970, which are bilaterally symmetrical across different elements of the body in a proximodistal direction, and the patterns are furthermore largely embedded within the keratinous scales, which are intact in their outline (Figures S3 and S4). There is also a clear dorsoventral variation in darkness. This conforms to expected distributions of color patterns, as have been observed in other fossils [13, 24, 26]. Exact hues will be difficult to determine [24], but we focus here on the relative differences in brightness and their likely adaptive function. Obviously, caution should be exercised when reconstructing vertebrate color patterns—particularly in reptiles, where colors can be varied dynamically using chromatophores rather than pigments locked into feathers or hair [24]. These dermal organs also may contain other pigments in addition to melanin, as well as protein conformations that produce structural colors (iridophores). Considering that the presence of chromatophores is a plesiomorphy of amniotes and amphibians [18] but was secondarily lost in birds and mammals [18] (birds still have chromatophores in their eyes [27]), these organs may well have been present in unfeathered dinosaurs. The loss of chromatophores is most likely due to the evolution of dense integumentary appendages, which rendered the chromatophores redundant in areas covered by hair or feathers [24]. Since it is unknown whether dinosaurs were primitively feathered [28], it is possible that unfeathered ornithischians could have retained chromatophores. However, most of the observed patterns in SMF R 4970 are clearly embedded within scales (Figures S3 and S4), which is incompatible with the pigments being incorporated into chromatophores [24].

### Pigment Patterns

**Figure 2:**
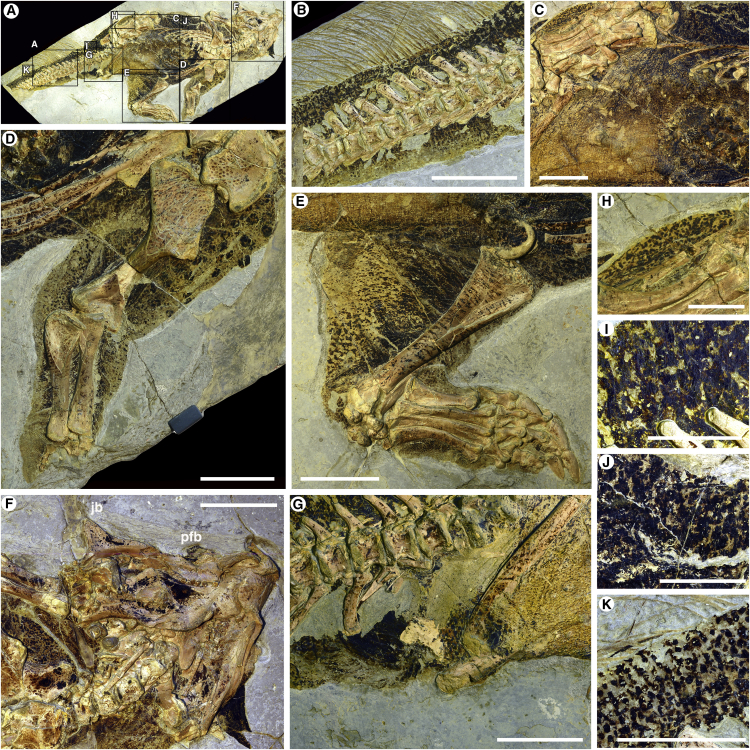
Details of *Psittacosaurus sp*. SMF R 4970, Photographed under Crossed Polarized Light Overview (A); tail region, showing countershading gradient (B); belly with lighter pigmentation (lower-left corner) and a dorsoventral pigmentation gradient (C); left forelimb with raised clusters of pigmented scales (D); left hindlimb preserving external disruptive patterns and striping on internal leg (E); head with patches of intensely pigmented integument (F); pigmented ischial callosity and cloacal region (G); integument associated with the right leg (H); detail of pigment patterns associated with the proximal tail region, dorsolateral surface (I); pigment patterns associated with the lateral torso (J); and pigment patterns associated with the distal tail region (K). Jb, jugal boss; Pfb, prefrontal boss. Scale bars represent 50 mm (B–G), 20 mm (H), and 10 mm (I–K).

There is a clear difference in pigmentation between the ventral surface on the lower belly and tail relative to the dorsum, which is more heavily pigmented (Figures 1 and 2; described in more detail in the Supplemental Experimental Procedures). The transition in dorsoventral pigmentation is well documented along the belly and the tail (Figure 2C). The chest is relatively more pigmented than the lower abdomen. Anastomosing pigmentation is present on the posterior section of the outward-facing integument of the hind leg and horizontal stripes on the anterior inward-facing surface; both resemble outline-breaking disruptive coloration [29, 30] (Figures 2E and S7). The more dorsal pigmentation varies from a dense (85%), dark pigmentation with dendritic, light-colored regions (Figures 2I and 2J) to a fine, spotted, lighter-colored pattern (60%–70% pattern density) on the distal tail (Figure 2K). The neck and head are more evenly pigmented, but the ventral surface exhibits lighter pigmentation, except for the face (Figures 2F and S8), where a black-pigmented region covers the area anterior to the orbitals, parts of the lower jaw, the jugal bosses, and prefrontal bosses (Figures 2F and S8). Certain exposed regions are more pigmented compared to adjacent regions, such as the ischial callosity (pubic sitting pad) (Figure S9), the ankles (Figure S8), and the scales covering the putative shoulder osteoderms (Figure S4), as well as the region around the cloaca (see the Supplemental Experimental Procedures; Figures 2G, S7A, and S9).

The distribution of the pigmentation patterns in SMF R 4970 suggests color patterns congruent with camouflage through background matching and countershading, in addition to some putative disruptive coloration on the legs, coupled with strong facial pigmentation suggestive of a signaling function and some protective/strengthening melanization.

### Reconstruction

**Figure 3:**
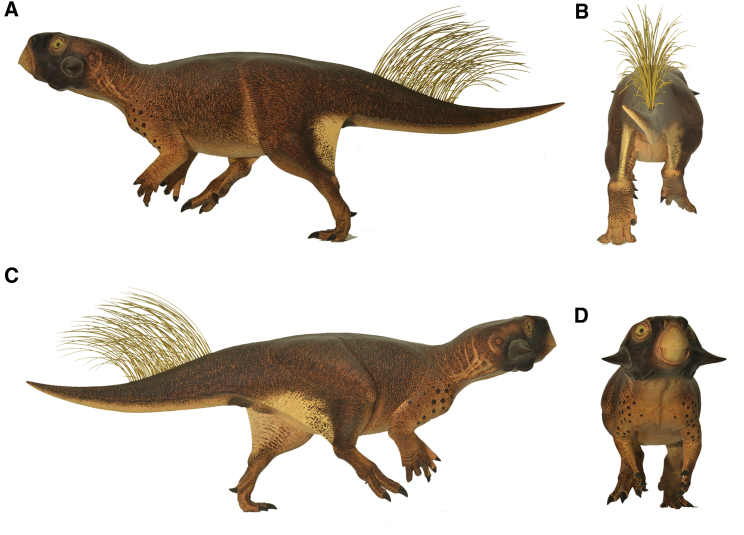
Model of *Psittacosaurus* Based on Skin and Pigmentation Patterns on SMF R 4970 Left lateral view (A), posterior view (B), right lateral view (C), and anterior view (D). See also Data S1 and S2.

**Figure 4:**
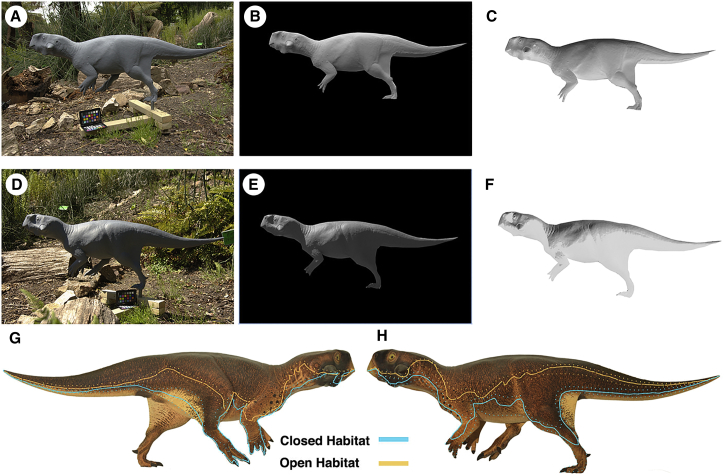
Testing *Psittacosaurus* Countershading in Natural Conditions (A–F) Gray colored cast without bristles attached, imaged under “closed habitat” conditions (A–C) and direct illumination (D–F). The model is shown as imaged in natural environment (A and D), masked (B and E), and in inverse color (C and F). (G and H) Predicted boundaries of rapid transition from dark to light skin for countershading in the diffuse illumination of closed habitats (blue) and of direct lighting in a sunny open habitat (orange). Stippled lines indicate 95% confidence intervals.

In order to interpret and further test the function of the observed color patterns, we produced a life-size model of *Psittacosaurus* SMF R 4970, carefully considering the volume and thickness of the body based on myoanatomical reconstructions and the preserved body outline (see the Supplemental Experimental Procedures and Figure S10). We produced two casts from this. Onto one model, we projected the observed color patterns. The similarity in color patterns between certain regions flanking the poorly exposed dorsal side allowed for extrapolation between these to produce a full body color reconstruction (Figure 3; see also Data S1 and S2). The second cast was painted uniform gray, to investigate the shadow that is cast in different lighting conditions [9]. By taking calibrated photographs under both diffuse and direct illumination in the field, we could calculate the surface reflectance that would nullify the gradients in reflected radiance [9] (Figures 4 and S17).

### Predicting Lighting Environment

The animal’s reconstructed patterns more closely match the predicted optimal countershading for diffuse illumination, as would be experienced in a “closed” light environment such as under a forest canopy [31]. The gradation from dark to light occurs relatively far down the body, unlike that which would be expected to optimize camouflage through countershading in the open, under direct sun [7, 9].

## Discussion

By comparing the observed distribution to that predicted for obliterating shape-from-shading cues under diffuse illumination, we infer that this small ceratopsian lived in a closed light environment such as under a forest canopy. Paleobotanical studies indicate that the lakes of the Jehol biota were surrounded by predominantly coniferous forest, with only a minor contribution from deciduous plants [32, 33, 34, 35]. The evidence for adjacent forests, such as petrified tree trunks in many of the lacustrine deposits yielding exceptional vertebrate fossils in the Jehol biota [36], and the dominance of evergreen canopy support our observations of adaptations for closed habitat illumination in this *Psittacosaurus*. It is, of course, possible that there was variation in color across the species’ range, and we would predict that this would mirror differences in the light environment.

Bristles on the tail [17] would have cast shadows that we did not model, but these shadows would not have provided cues to the 3D shape of the animal and, being vertical and somewhat flexible, could not have been nulled by countershading. Other patterns are of potential significance: the strongly colored face (Figure S8) resembles masks seen in different mammals. The functions of these are still debated, including aposematism, anti-glare, dazzle, conspecific signaling, and thermoregulation [37]. It is worth noting that the jugal bosses in ceratopsians have been suggested to have had a signaling function [38]. We note that the heavy pigmentation in the face is not locked into scales and could thus have been involved in a more colorful display through chromatophores also. We interpret the pigmentation of the scales on the ankles and ischial callosity as strengthening melanization [39]. It is also observed that melanin serves in putative antimicrobial defense for organs, including the anus and urogenital system of tetrapods; this would explain the cloacal pigmentation [40]. The disruptive anastomosing patterns and striping on the hindlimbs is known in some modern forms. In addition to zebras, the African donkey and the more distant okapi exhibit striping on their limbs. These patterns have received the most attention in zebras, in which stripes were attributed to predator and/or parasitic fly avoidance and grooming behavior [41, 42, 43, 44].

It has been shown that countershading also correlates with positional behavior, as is observed in many species of caterpillar that live upside down and exhibit reverse countershading [45, 46]. In primates too there is a strong correlation between countershading and positional behavior. The observation that the chest in SMF R 4970 is relatively more pigmented than the belly and tail support osteological evidence for bipedalism in adult psittacosaurs [47], but correlation of pigmentation to bipedalism needs more scrutiny.

More speculatively, we suggest that the predators of this species most likely used shape-from-shading cues in detecting prey, given that optimized countershading obliterates this information [7]. The likely predators were probably the larger theropod dinosaurs of the Jehol Biota. Modern birds have a well-developed optic lobe, which sees its origin in maniraptoran dinosaurs along with an expansion of the cerebrum, which has been correlated to the evolution of flight [48] and the need for more accurate depth perception. Binocular vision becomes more prevalent in coelurosaurian dinosaurs [49], again related to depth perception. It is not well known how widely shape-from-shading is utilized in the animal kingdom outside of humans. Evidence from pigeons [50] and cuttlefish [51] amounts to their use of the same visual cues and the convergent evolution of these. However, the strong adaptive evolution of countershading in both aquatic and terrestrial realms speaks toward its broad utilization in vertebrate predators. Fossil countershading is known from fossil fish from a variety of sites, ranging back to at least the Permian or Carboniferous [24], whereas this psittacosaur was the first terrestrial fossil to have been shown to be countershaded [16]. Therefore, the opportunity that such exceptionally well preserved specimens creates for reconstructing coloration may illuminate our understanding of not only the habitat, but also the visual cognition of the animals within it.

## Experimental Procedures

### Dinosaur Color Pattern Analysis

The well-preserved specimen of *Psittacosaurus* sp. held at the Senckenberg Museum, SMF R 4970, was studied in the public exhibit (see the Supplemental Experimental Procedures for information on its taxonomic status). The specimen was photographed with the protective glass removed using a 800 W tungsten light source with a crossed polarized light configuration. In addition, LSF imaging was used to reveal colored scale patterns in the darker zones of the specimen (for more information, see the Supplemental Experimental Procedures). Small samples of the integument were taken from a few places for electron microscopy analysis (for more information, see the Supplemental Experimental Procedures).

### Reconstruction

The preserved skeleton was measured and used to produce a 1:1 anatomical model of SMF R 4970. Cranial details were obtained from a 3D printed skull of a different specimen. Volume was added to the skeleton based on knowledge of major muscle groups and the preservation of integument outlines of the specimen. Decay has not lead to distortion of the integument relative to the skeleton, which suggests rapid burial after deposition and little prior rot to the animal before it sank to the bottom of the lake. Exposed scale patterns were projected onto the model and extrapolated between unexposed regions. Exposed color patterns were similarly carefully projected onto the model and extrapolated across unexposed parts based on similar patterns, assuming bilateral symmetry between left and right patterns as confirmed in certain parts (for more information, see the Supplemental Experimental Procedures).

### Predicting Lighting Environment

In order to predict the environment in which the observed countershading was optimal, we took a model, painted uniformly gray, and photographed it under different lighting conditions; we chose a sunny and a cloudy day and photographed the specimen in an open and a closed habitat. The model was cropped and the image inverted in order to produce the optimal countershading patterns under each circumstance in order to compare with the observed patterns in the fossil (for more information, see the Supplemental Experimental Procedures).

## Author Contributions

J.V. conceived the study. J.V., R.N., M.P., T.G.K., and G.M. studied the color patterns and taphonomy. R.N. modeled and painted the dinosaur. S.L. provided additional 3D models and figures. E.R. and S.L. supervised the anatomical modeling. I.C.C. and J.V. did the calibrated photography. S.L. did the 3D photogrammetry. T.G.K. and M.P. performed the LSF imaging. I.C.C. did the visual modeling. J.V. and I.C.C. wrote the paper with comments from all authors.

## Supplemental Information

Document S1. Supplemental Experimental Procedures and Figures S1–S17 Document S1. Supplemental Experimental Procedures and Figures S1–S17

Data S1. 3D Photogrammetric Representation of *Psittacosaurus*, Related to Figure 3 Data S1. 3D Photogrammetric Representation of Psittacosaurus, Related to Figure 3

Data S2. 3D Photogrammetric Representation of *Psittacosaurus* Indicating Preserved Regions, Related to Figure 3 Data S2. 3D Photogrammetric Representation of Psittacosaurus Indicating Preserved Regions, Related to Figure 3

Document S2. Article plus Supplemental Information Document S2. Article plus Supplemental Information
