# Supplementary figures and images for: 3D Camouflage in an Ornithischian Dinosaur
Source: Curr Biol. 2016 Sep 26;26(18):2456–62. doi: 10.1016/j.cub.2016.06.065 (PMC5049543; doi:10.1016/j.cub.2016.06.065)

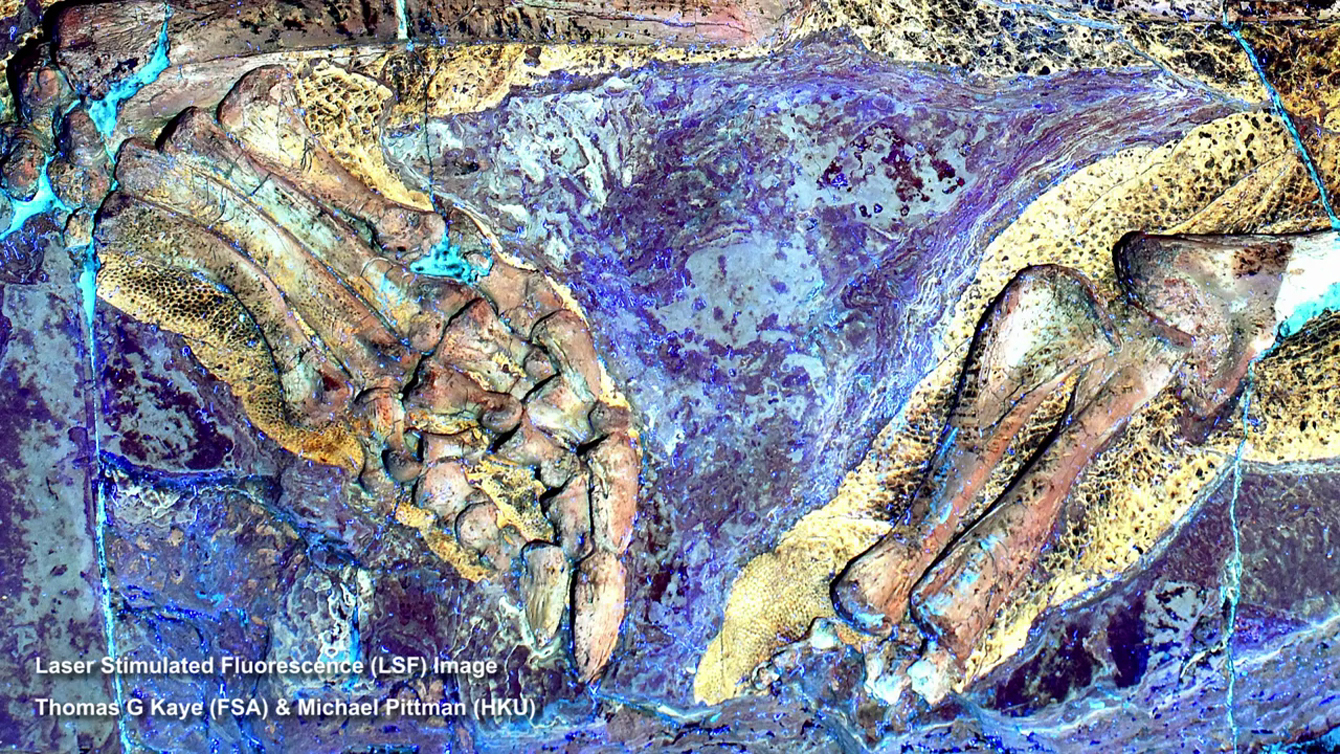

Supplement: Supplementary file 1 [file mmc5.jpg]

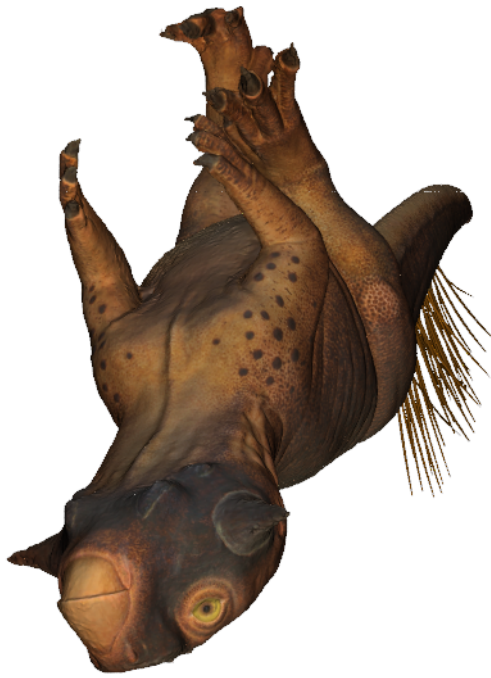

Supplement: Data S1. 3D Photogrammetric Representation of Psittacosaurus, Related to Figure 3 [file mmc2.pdf]

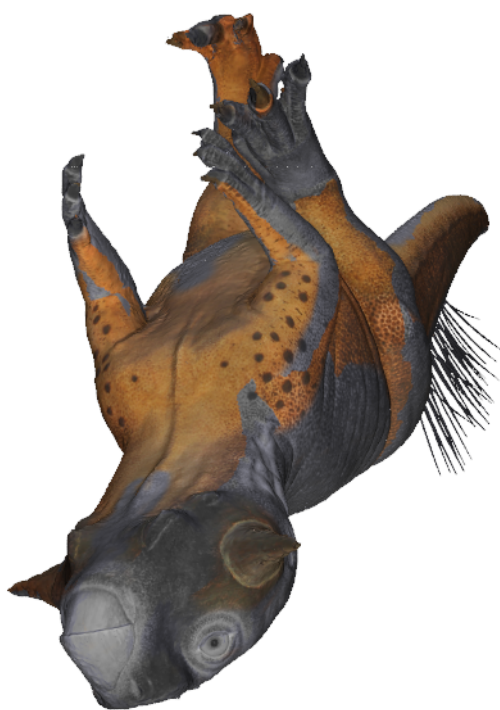

Supplement: Data S2. 3D Photogrammetric Representation of Psittacosaurus Indicating Preserved Regions, Related to Figure 3 [file mmc3.pdf]
