# Supplementary material for: 3D Camouflage in an Ornithischian Dinosaur
Source: Curr Biol. 2016 Sep 26;26(18):2456–62. doi: 10.1016/j.cub.2016.06.065 (PMC5049543; doi:10.1016/j.cub.2016.06.065)
Supplement: Document S1. Supplemental Experimental Procedures and Figures S1–S17 [file mmc1.pdf]

Current Biology, Volume *26*

Supplemental Information

3D Camouflage in an Ornithischian Dinosaur

Jakob Vinther, Robert Nicholls, Stephan Lautenschlager, Michael Pittman, Thomas G. Kaye, Emily Rayfield, Gerald Mayr, and Innes C. Cuthill

Supplemental Experimental Procedures

Description of specimen

Preparation

Taxonomy

Imaging techniques

Soft tissue visual enhancement through crossed polarised photography

Laser-stimulated fluorescence (LSF) imaging

Taphonomy

Integument taphonomy

Integument versus internal organ melanin

Compression and distortion

Pigment patterns across body

Countershading

Dorsal pigment patterns

Hindlimb

Forelimb

Face and neck

Structural and protective pigmentation

Reconstruction of *Psittacosaurus* SMF R 4970

Keratinous sheaths

Volume rendering

External surface

Modelling process

Digital model

Predicting countershading

Description of specimen

Preparation.

The specimen of SMF R 4970 arrived at Senckenberg partially prepared (Figure S1). The remainder of the soft tissues was further exposed, using pneumatic tools and finally sandblasting with dolomitized chalk. To protect the bones and soft tissues, the surface was varnished.

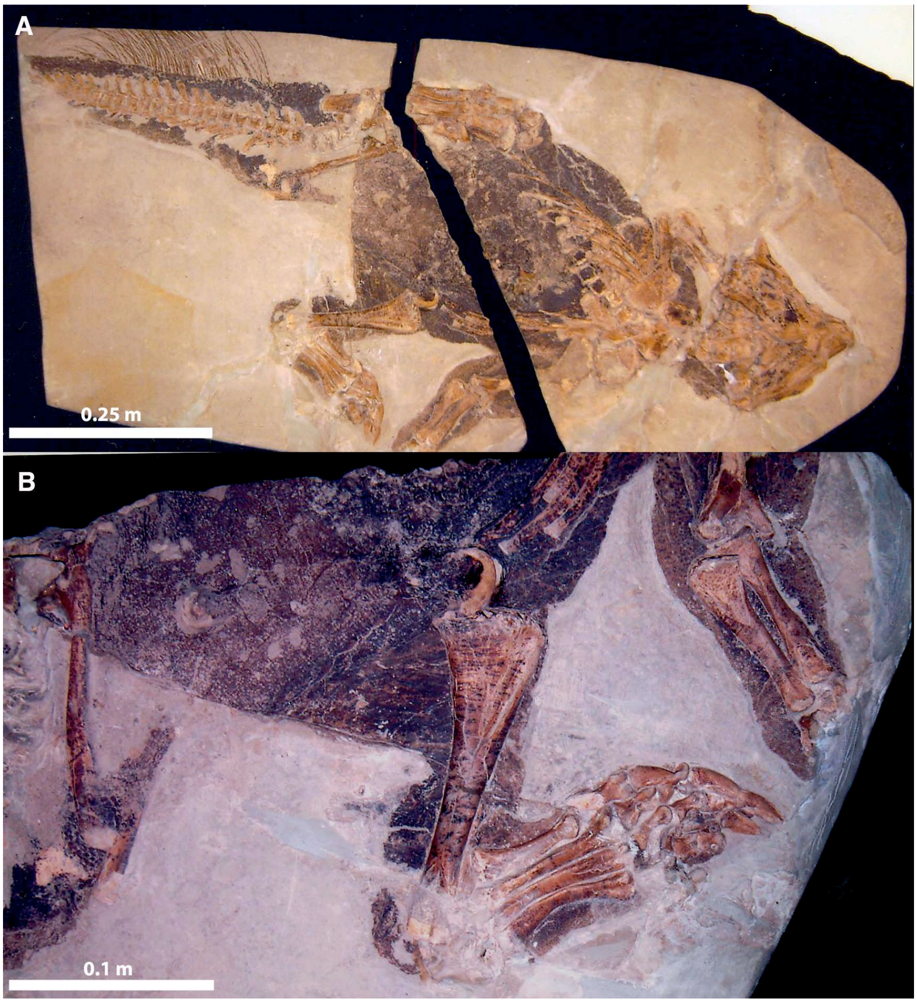

Figure S1. *Psittacosaurus sp.* SMF R 4970 prior to preparation. (**A**) whole specimen, (**B**) detail of left hind and forelimb as well the ischial region. Photos, courtesy of Olaf Vogel.

Taxonomy

According to Sereno (Fig. 2.1) [1] there are four species of *Psittacosaurus* known from the Jehol Biota of Liaoning Province: *P. meileyingensis*, *P. lujiatunensis*, *P. major* and *P. mongoliensis*. None of the autapomorphies he proposed for them - except for one for *P. mongoliensis* - can be verified in SMF R 4970, mostly

because they are cranial features that are not visible in the ventral view that the skull is preserved in. SMF R 4970 is not *P. mongoliensis* as it lacks a transverse expansion of the distal end of the ischial blade that is twice its mid-shaft width (Sereno 2010: autapomorphy 2). SMF R 4970 does have a hypertrophied dentary flange – proposed as an autapomorphy of *P. major* (Sereno 2010: autapomorphy 5) – but this feature is also known from the holotype and paratype of *P. lujiatunensis* [2]. Hedrick & Dodson [2] proposed that *P. meileyingensis* and *P. major* are both junior synonyms of *P. lujiatunensis* using 3D geometric morphometrics. They suggested that the hypertrophied dentary flange is instead an ontogenetically-related feature of *P. lujiatunensis* [2]. Thus, in the absence of observable autapomorphies of *P. meileyingensis*, the absence of a *P. mongoliensis* autapomorphy and the taxonomic status of *P. lujiatunensis*, we refer SMF R 4970 to *Psittacosaurus* sp. in this study.

Imaging techniques

Soft tissue visual enhancement through crossed polarised photography

Due to the varnish and in order to avoid glare off of the surface when documenting the pigment density across SMF R4970, the specimen was illuminated using a 800 W tungsten light source (Lowell totalight) with a linear polarising gel mounted in front of it. The specimen was then photographed, using a Nikon D800 on a tripod, with a 60mm macro lens and a polarising filter attached. White balance was set 3200 K, image quality to ISO 100 and photographed in TIFF (5520 × 3680 pixels). The polarising lens filter was rotated to exclude the subjective maximum (polarised) glare from the surface [3]. Images are shown in Figures 1, 2, S2, S4, S5, S6, S7.

Laser-stimulated fluorescence (LSF) imaging

Laser-stimulated fluorescence (LSF) imaging is a versatile observational technique that has a multitude of paleontological applications [4]. SMF R 4970 was scanned with a custom device (KayeT Scanner 3.0) that has been developed significantly since originally presented [4] (see Figures S2). Here a 405

nanometer, 500 milliwatt violet laser was interfaced with a Powel line lens that projected an even vertical line vertically across the specimen. The line was scanned horizontally by a motor assembly across the specimen. Scanning was performed in a dark room while a time exposure was taken through a tripod mounted Nikon D810 camera. A 425 nm long pass filter was employed in front of the camera lens to block the laser light. Images were post processed to equalize colour representation using Photoshop, MaximDL and Image Lab. Proper precautions using laser-blocking protective glasses and manufacturer's safety protocol were followed.

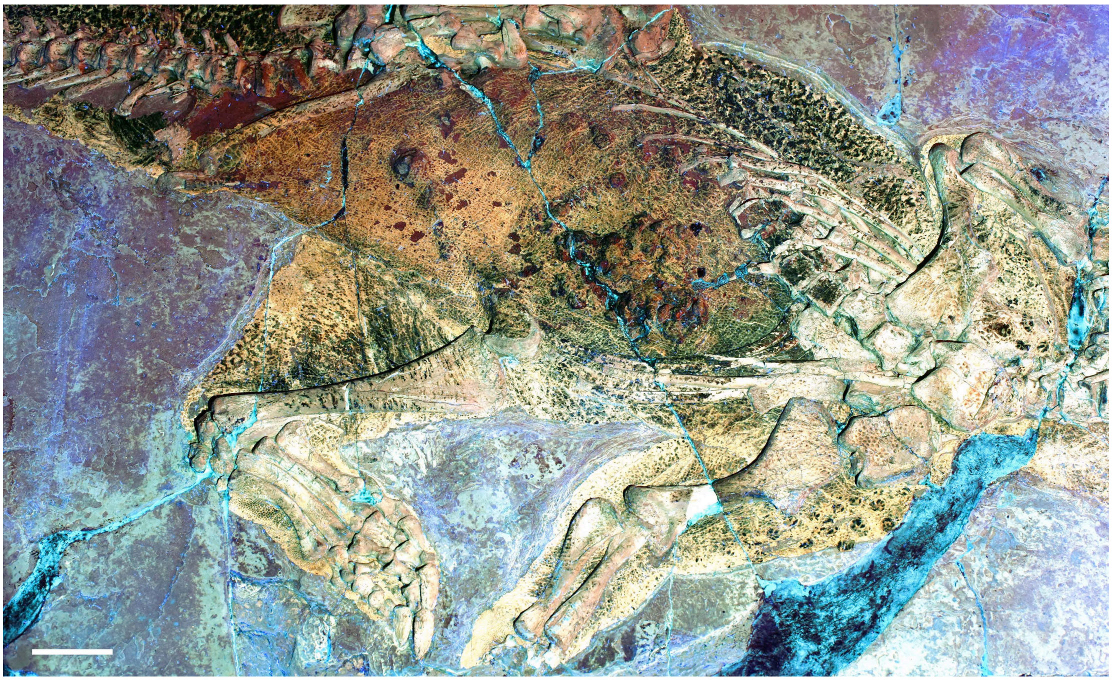

Figure S2. *Psittacosaurus sp.* SMF R 4970 imaged with laser stimulated fluorescence. Scale bar 50 mm.

Taphonomy

The specimen, SMF R4970, is compressed with the ventral surface exposed in an oblique lateral view. Integument is preserved across the whole body as a two-dimensional film and superimposed on bones. Some integument has been prepared away from bones or has worn off. Organic material within the abdomen is clearly visible under crossed polarised light and interrupts the trajectory of the ventral integument across this region. LSF imaging, however, demonstrate that the ventral integument continues across the abdomen (Figure S3G, S6). Fluorescence of the lightly pigmented scales is visible under laser light

overlying the pigmented internal organs (Figure S3G, S6) (see section: *Integumentary melanin vs. internal organ melanin* below). The integument preserves as a carbonaceous/calcium phosphate film, which varies from a buff/greenish yellow to black/brown in colour under crossed polarised light. Scales of varying density and shapes are clearly visible across the body, also evident with LSF imaging (Figure S3).

Keratin taphonomy. LSF imaging show clear fluorescence of scales across the body of the specimen. It is well documented that keratinous structures are often enforced with calcium phosphate salts [5], which is the likely source of fluorescence observed across the specimen as is commonly observed in preserved claw sheaths and feather rachises [6, 7]. The trace amount of calcium phosphate would provide the modest relief that enables the ability to discern scales, even when unpigmented.

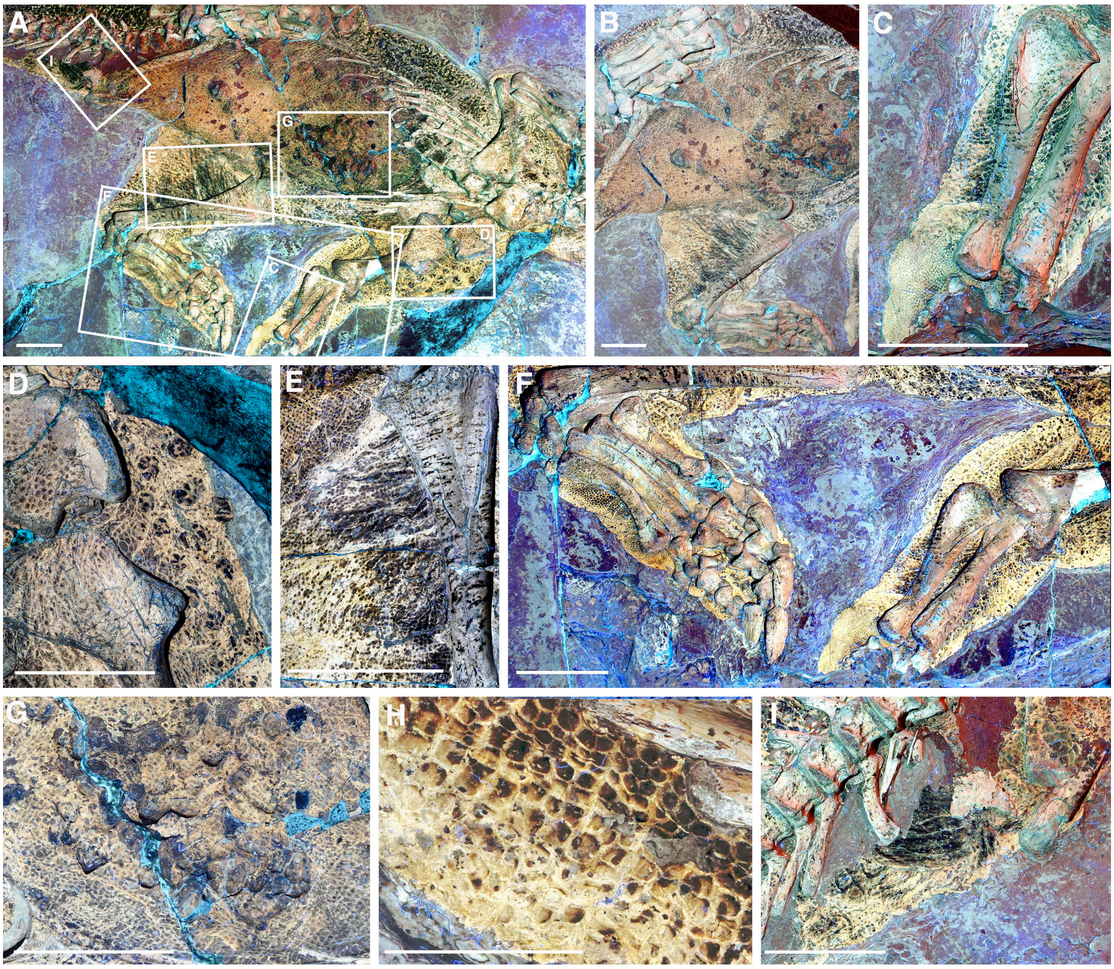

Figure S3. ***Psittacosaurus sp.*** **SMF R 4970 Details under laser stimulated fluorescence.** (**A**) Whole specimen with indications of details in following

panels. (**B**) Abdomen and hind limbs. (**C**) Left lower arm and wrist. (**D**) Left shoulder. (**E**) Left hindlimb with details of conflated external and internal integument. (**F**) Left forelimb and hind limb. (**G**) Detail of integument superimposed over gastroliths in abdominal section. (**H**) Ventral tail integument with countershading gradient shown. (I) Ischial callosity and cloacal region. Scale bars 50 mm, except for H, which is 10 mm.

Melanin taphonomy. Studies have shown that melanin preserves commonly in exceptional fossil localities [8, 9], including the Jehol Biota [10-12]. The observed differences in brightness are here surmised to be related to differences in original melanin density. Other important integumental molecules, that have been thought to preserve, are keratin [13, 14] and collagen [15, 16]; both are protein molecules. Convincing evidence of organic residues derived from proteins are known from mollusc shell matrices [17], which shows that incorporation into a mineralized matrix provide a stable environment for long term stability [18], but proteins do not survive in the presence of fluids, thermal diagenesis, nor bacterial activity. This can be ascribed to the varied thermal stability of amino acids, weakness of the peptide bonds and bacterial consumption prior to encapsulation. Indeed, no evidence of keratin has been observed in the many studies of fossil feathers [8, 19-21].

It is observed in SMF R 4970 that the scales are delimited by outline and some topography, but the darkness of the imprint is not correlated with these, which suggests that this is embedded pigment within the scales rather than the keratin (Figure S4). The degree of fluorescence of the keratin phosphate salts provide a rough guideline for the original outline and thickness of the scales. While collagenous material has been reported from animal integuments [15], these studies are not based on chemical evidence, but from structural comparisons, which are hard to confirm. Some of these structures are melanin-bearing proto feathers [12, 22]. Alternative compounds potentially responsible for the observed impressions are residual lipids, which are known to fossilise well [23]. The presence of clear fluorescence in the preserved scales are likely to be responsible for the slight colour change in relation to the matrix and the unpigmented scales rather than any organic residues.

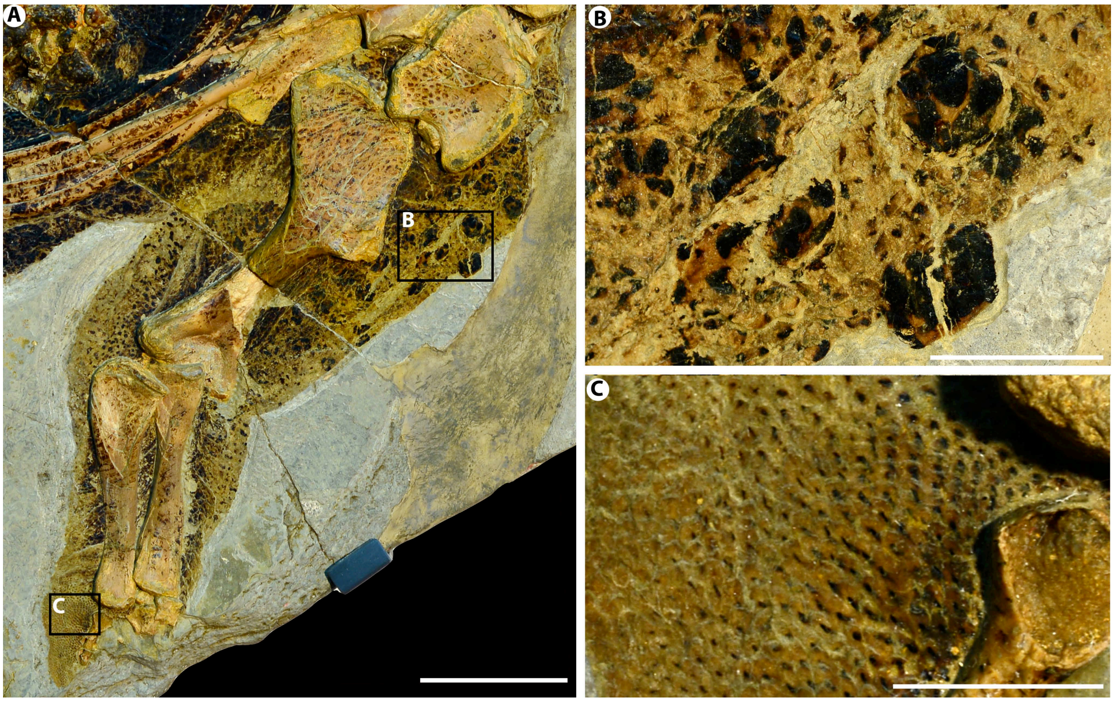

Figure S4. ***Psittacosaurus sp.*** **SMF R 4970.** (**A**) Left forelimb, (**B**) detail of the shoulder/chest and raised, enlarged pigmented scales covered by smaller scales, (**C**) scale covering the finger pad, showing discrete pigmentation within scales. Crossed polarised light. Scale bar in (A) 50 mm, (B), (C), 10 mm

Studies have shown that colour patterns preserved in fossil feathers are due to the original distribution of melanosomes, preserved in the fossils and which is the only carbonaceous residue in the fossil leaving a colourised imprint [8, 24]. Relative colour intensities in fossil feathers are also correlated with melanosome densities, as in modern birds [24]. While few studies have investigated the effect of melanin to observed colours in animal skin, the clear colour patterns and evidence for embedding within keratinised scales suggest that the organic imprints are a reliable proxy for the original relative colour patterns across the body, and that chromatophore complexes [25] were not present in any major part of the body.

Melanosome microbodies

Due to the varnish cover on the specimen, sampling was complicated. However, powder sized chips were taken using a scalpel from four regions of the body with

different degrees of organic material content. These were mounted to an SEM stub with carbon tape.

Similar to other specimens from Jehol [10], melanosomes are preserved as either microbodies or impressions in the matrix. Quartz crystals, in particular, preserve aggregates of melanosome-like microbodies (Figure S5A,B). These microbodies are ovoid in shape, about 400 nm in length and about 250 nm in width (n=15), which suggest a phaeomelanin rich melanosome [10, 11]. Quadratic Discriminant analysis predict a brown colour (p=0.9982), clustering with modern reddish brown feathers among brown samples. This is similar to observations from two other psittacosaur specimens [26]. Together with the clear macroscopic colour patterns, which show bilateral symmetry and a clear dorsoventral gradient, this provides evidence for melanin based colouration. Recent papers have advocated that bacteria could produce the same structures and are an equally parsimonious interpretation [27]. However, bacteria are much more varied in morphology. Naturally occurring bacteria are not shown to concentrate in melanin-bearing tissues to the exclusions of others and to exhibit such limited morphologies as is seen in melanosomes [9]. A recent study has tested the melanosome versus bacteria hypothesis by chemical analysis using time-of-flight secondary ion mass spectroscopy (TOF SIMS) of fossil organic soft bodied tissues ranging in age from Carboniferous to Miocene and included tissues, such as eyes, hair, feathers, skin and ink sacs from organisms, spanning cyclostome fish, amphibians, birds, mammals and cephalopods. All fossil taxa yielded signatures consistent with diagenetically altered melanin. A similar study has found melanin in the slightly older *Anchiornis huxleyi* from China [28].

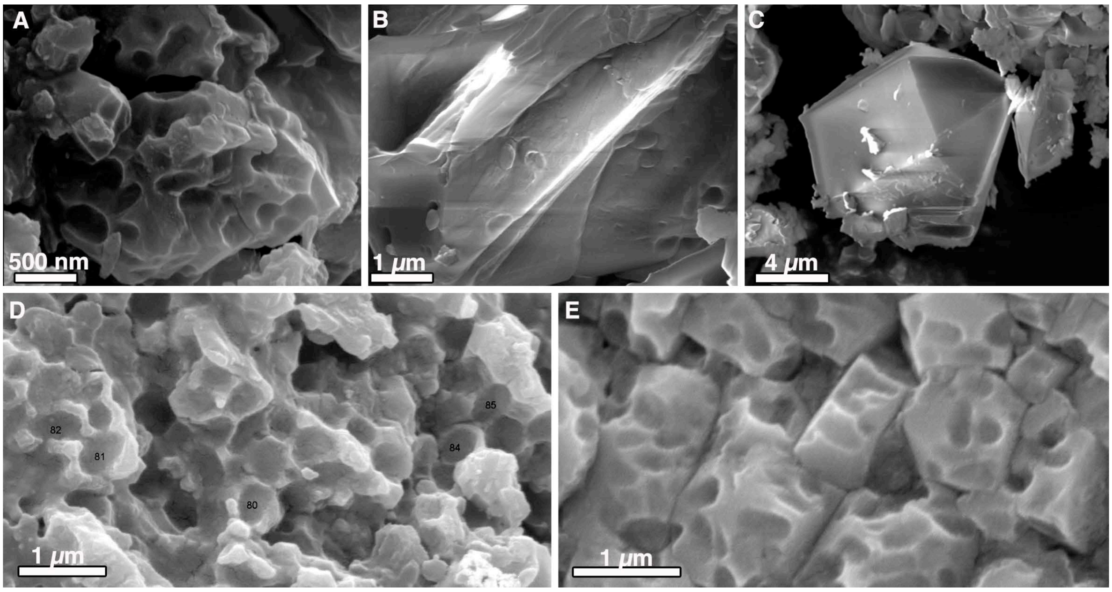

Figure S5. ***Psittacosaurus sp.*** **SMF R 4970 organic residues, preserving melanosome microbodies and comparisons with two other psittacosaur specimens.** (**A**) Quartz grain with dense and regularly shaped oblong microbodies interpreted as original melanosomes due to their shape conforming to phaeomelanosomes and similar microbodies in two other psittacosaur integuments [26]. (**B**) Another quartz grain preserving less dense melanosome microbodies as threedimensional bodies and as impressions. (**C**) A quartz crystal lacking melanosome impressions from outside of the preserved integument. (**D**) Melanosomes from *P. lujiatunensis* (Peking University V1050) and (**E**) *P. lujiatunensis* (PKUP V1051). (**D**, **E**) image courtesy of Li Quanguo and Matthew Shawkey. Scale bars indicated in figure.

Thus, evidence for these structures being melanin-bearing melanosomes are supported by the preservation of visible colour patterns, localisation of melanosome-like microbodies within tissues that are known to contain melanin and chemical analyses of similar microbodies in other taxa. Bacteria, on the other hand, exhibit a greater morphological range and are present in all tissues during decay. Furthermore, geobiological studies show that bacteria need more exceptional circumstances to be preserved, through encapsulation within, for example, synsedimentary chert. The observed quartz crystals in the *Psittacosaurus* specimen are clearly late diagenetic, present as euhedral forms,

scattered in the rock. Quartz crystals outside of the integument do not preserve microbodies, for example (Figure S5C).

Integumentary melanin vs. internal organ melanin.

Melanin-bearing melanosomes are present not only in the integument and integumentary appendages, but also eyes, certain nervous tissues and many internal organs - especially the liver. As an example, the holotype of *Sinosauropteryx* NIGPAS 127586 [29] preserves a well delineated liver as well as the retinal melanin of the eye. It is therefore pertinent to discriminate between these when evaluating original external pigment patterns. We observe that SMF R 4970 preserves gastroliths and a darker and thicker layer of organic material is associated with it, particularly in the anterior section of the abdomen. Under crossed polarised light (Figure S6A,B) this organic stain is seen to be superimposed on some left ribs as well as vertebrae, which negates it being dorsal integument exposed. Ventral integument is clearly delineated and can under LSF be seen to extend across the gastroliths due to the fluorescence of the scales covering the region, which are otherwise indistinct due to the darkness of the underlying organic material (Figure S6C,D, S3G). Similarly, there is distinct scaly integument superimposed on the humerus, coracoid and scapula, which is clearly ventral integument and less melanised. We thus treat the dark abdominal patch (Figure S4) as non-integumentary melanin, and potentially plant-based food contents that has to be ignored for external integumentary reconstruction.

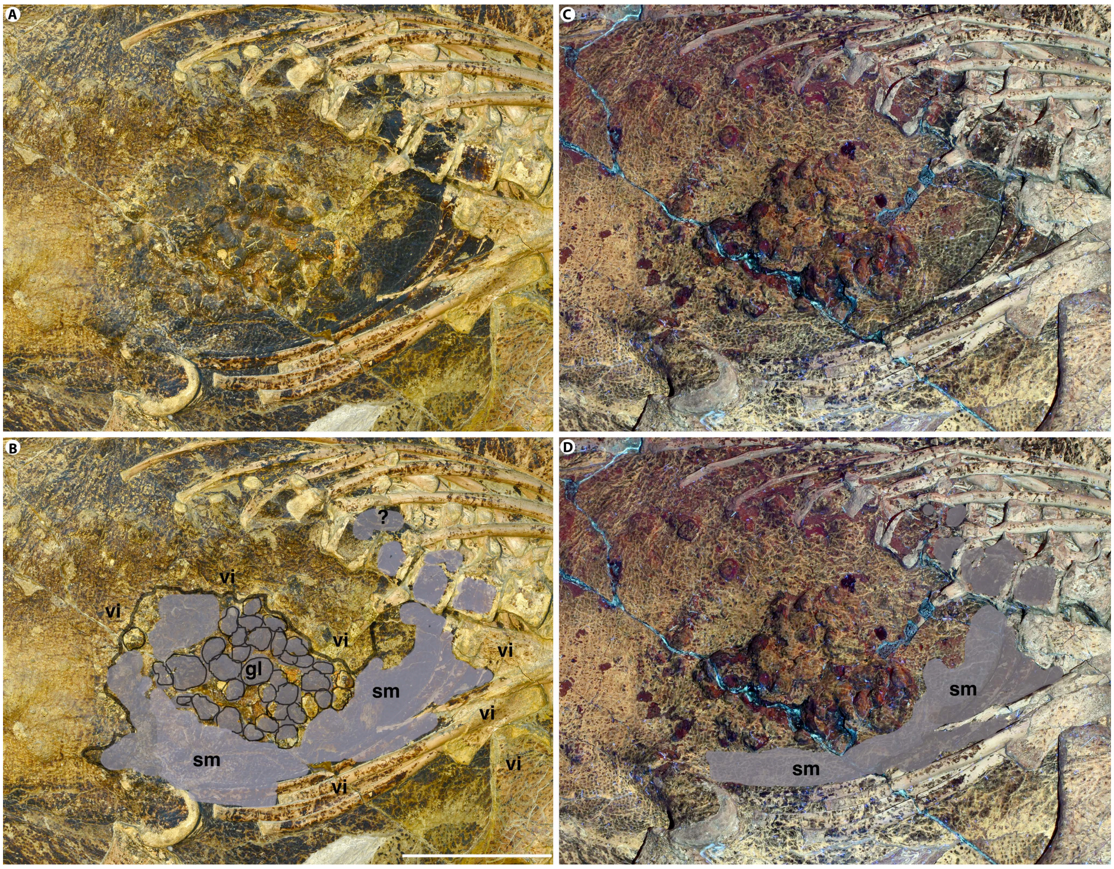

Figure S6. ***Psittacosaurus sp.*** **SMF R 4970.** (**A**) Abdominal region imaged with crossed polarised light, (**B**) same region as in (A), but with organic material derived from internal organs demarcated. (**C**) Same region as in (**A**) photographed using LSF showing fluorescing keratinous ventral integument overlying stomach. Abbreviations, ventral integument (vi), visceral/abdominal melanin: "stomach melanin" (sm), gastroliths (gl). Scale bar 50 mm.

Compression and distortion. It has been shown that compression fossils do not exhibit any lateral distortion or widening during the process of flattening [30] and thus that a fossil can be more or less interpreted as a 2D compression of a 3D view. This pertains also to SMF R 4970. However, decay prior to burial and compaction can lead to distortion of the body. The skeleton remains in perfect articulation, and there is no obvious distortion to the integument, which both suggest rapid burial and encapsulation. However, while the body appears in an oblique ventral view, the tail vertebrae seems to be displaced laterally, as is evident by the proximal displacement of the tail vertebrae, which appear to be in a lateral view. The integument appears to be in an oblique ventral view as the

dorsal quill filaments on the tail extend below the exposed dorsal margin about 4 cm. The ventral midline is delineated by the preserved cloaca and ischial callosity.

Pigment patterns across body

Countershading. A visible difference in brightness is present on the ventral surface relative to the dorsum. On the tail, a transition goes from an unpigmented ventral surface to a surface that is pigmented on the lower lateral side in a transition close to the narrow band of unpigmented ventral surface. Thus the transition would have been situated on a ventrally facing flank of the tail (Figure 2b).

The belly is similarly lightly pigmented, grading gradually (Figure 2C) to a more pigmented surface, which appears similar to the dorsalmost exposed surface in pigmentation around the lower lateral side of the belly. The chest is relatively more pigmented and is covered by more prominent scales (i.e. on the coracoid and humerus) than the belly (Figure 2D).

Dorsal pigment patterns. The dorsal surface is not well exposed, but dorsal and lateral surfaces are seen along the body in several places, through the postero-orbitals, laterally on the torso, and the dorsal tail (Figure 2K). The dorsal pigment patterns seen through the postorbitals of the skull consist of a fine pattern of homogenous pigmentation with a subtle variation in brown patterns on a millimeter scale.
On both sides of the torso, dense pigmentation is seen (Figure 2J), which consists of black patches (~85% coverage, assessed using a Rite-in-the-Rain geology chart of grain density) with interdigitating unpigmented and dendritic or irregular anastomosing patches. This pattern continues across the torso and is also visible on the dorsal tail (Figure 2I). The distal tail possesses a less dense pigment pattern with digitate circular patches (60-70% coverage) (Figure 2K).

Hindlimb. The left hindlimb is well exposed on the tibia-fibula and the pes (Figure S7A). The observed patterns behind the tibia-fibula are a combination of

internal and external integument. Superimposed on the tibia is an integument devoid of scales, which shows horizontal pigmented stripes, becoming denser and thinner proximally. The stripes can be traced posteriorly, where they are superimposed on the external integument, which is scale clad. The external integument is densely pigmented, confined to distinct scales proximally and anteriorly, but is becoming less dense posteriorly with only about 5% density of spots at the posterior-most margin, while the posterior patterns closer to the ankle joint consist of an anastomosing/reticulate pattern of dark pigmentation, which appear to be unconfined to scales. Large scales cover the ankle and the upper metatarsals, while finer scales cover the lower metatarsals and phalanges. These are all pigmented. The right hindlimb is compressed vertically (Figure S7B). Some integument of the external surface of the leg exhibits pigmented scales with an anterior-posterior gradient in pigment density. Integument from the anterior surface of the thigh is seen as a compressed layer of imbricated folds of integument. The thigh possesses a denser pigmentation than the leg with about 70-80% density of pigmentation.

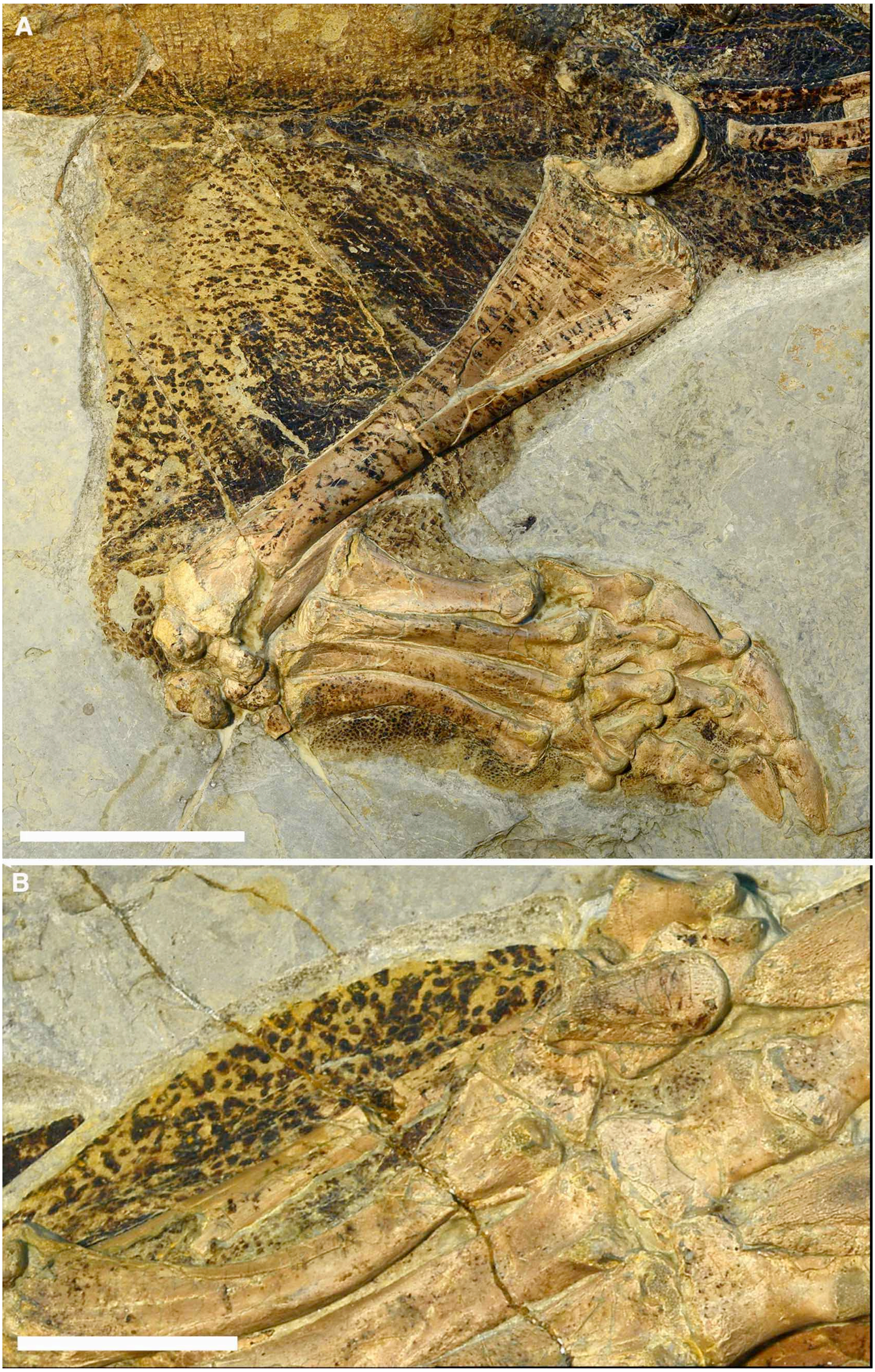

Figure S7. ***Psittacosaurus sp.*** **SMF R 4970 hindlimbs**. (**A**) Left hindlimb, (**B**) right hindlimb. Crossed polarised light. Scale bar in (A) 50 mm, (B) 10 mm.

Forelimb. The integument on the chest and shoulders (on top of the coracoid and proximal humerus) is covered by relatively large pigmented scales, covering ~50% of the integument. The integument anterior to the humerus preserved in the specimen shows clear circular patches consisting of a number of pigmented scales, which exhibit some relief. These structures could indicate underlying osteoderms overlain by multiple scales, although no bone appear associated with them and they could as well be a raised association of larger pigmented scales without an underlying osteoderm. The adjacent integument exhibits sparser scales, which are more lightly pigmented. The pigmentation density is highest laterally in the integument superimposed on the humerus and ulna/radius (~80%), while the anterior and posterior integument is lighter pigmented, decreasing to <10% pigmentation density. The only well-preserved fingerpad is covered by minute scales in which discrete depositions of pigments are observed (Figure S4C). The shape of the pigment spots within each scale varies from a line to a rounded spot and can be situated centrally to marginally.

Face and neck. Much of the integument has been prepared, or has been weathered away on the skull bones (Figure S8A). However, distinct patches of pigmentation can be characterized between different areas. A very thick layer of pigmentation with a black appearance is seen across the right and left dentary (Figure S8B). The heavy pigmentation continues ventrally and is superimposed also on the splenial. Dorsally, it transcends across the dentary and maxillary dentition to the jugal horn and up to the prefrontal horns (Figure S8C, F). Lighter pigmentation is seen anteriorly, on the predentary. The pigmentation on the predentary appears to form a lighter layer, while the rostral appears to have been prepared to a level in which potential pigmentation is lost. Dorsal pigmentation is seen through the inferior temporal fenestrum on both sides of the skull and consists of a lighter, but relatively dense layer with small scales (Figure S8E). Neck pigmentation is seen on the left side of the skull superimposed on the neck vertebrae and draping over the quadrate. The skin comprises here regularly sized scales with variable pigmentation, forming oblique stripes. Dorsal skin is observed on the right side of the neck where the specimen has been prepared to a lower level in the rock. A distinct break of slope

between the shoulder and this integument is seen (Figure S8D). The integument here is similar to the ventral neck observed on the left side in terms of scale morphology and size, but is more densely pigmented.

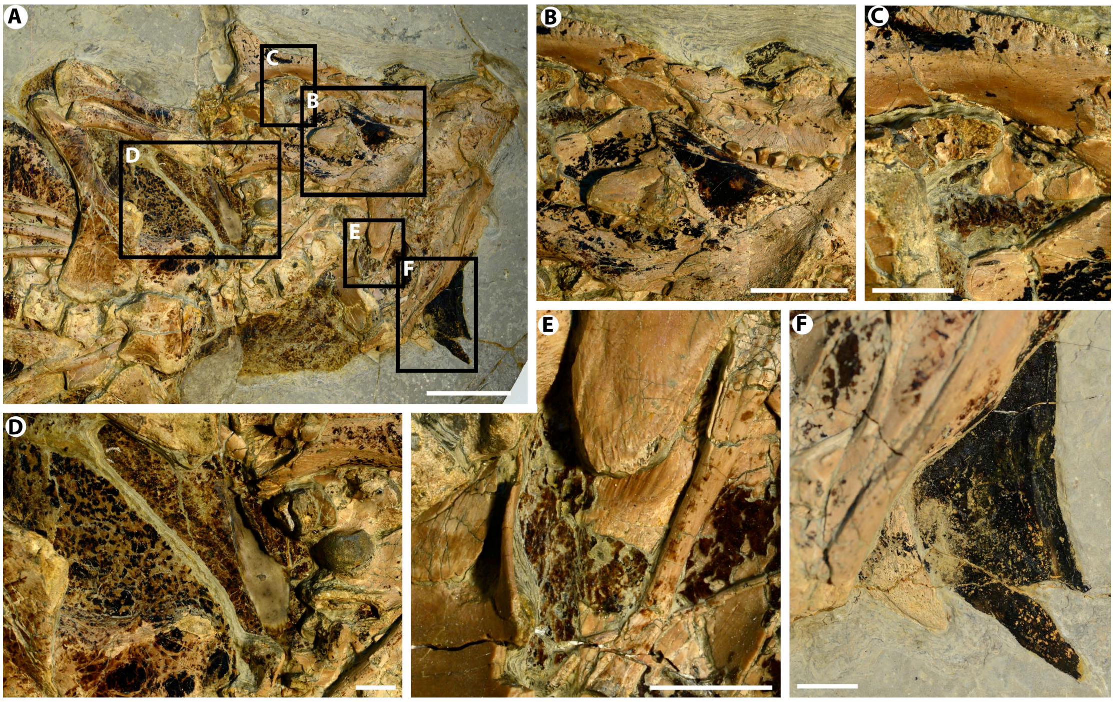

Figure S8. *Psittacosaurus sp.* SMF R 4970 skull detail. (**A**) Overview, (**B**) right dentary and mandibular region in ventral view, (**C**) , right jugal in ventral view, (**D**) shoulder region in ventral view, (**E**) quadrate and temporal region in ventral view, (**F**) left jugal horn in ventral view. Scale bar in (A) 50 mm, (B) - (F) 10 mm.

Structural/protective pigmentation. Melanised keratinous integument has been shown to be more resistant to abrasion [31]. It is seen that melanised feathers are present in the distal primaries of the otherwise white seagull and that bird bills are harder and more abrasion resistant when melanised [32]. Several tissues are also melanised for antimicrobial reasons, such as the anus, scrotum as well as internal organs [33], such as the liver [34]. The integument over the distal ischium exhibits larger scales than those on the adjacent ventral integument, which are also heavily pigmented. This region is likely to have formed a resting pad, or ischial callosity, when crouching. These areas would have needed additional protection. The cloaca, which is inferably posterior to the pubic bone, is also heavily pigmented, forming a large patch, whose outline is

obscured by rock breakage (Figure S7). This is likely to have been present for antimicrobial protection.

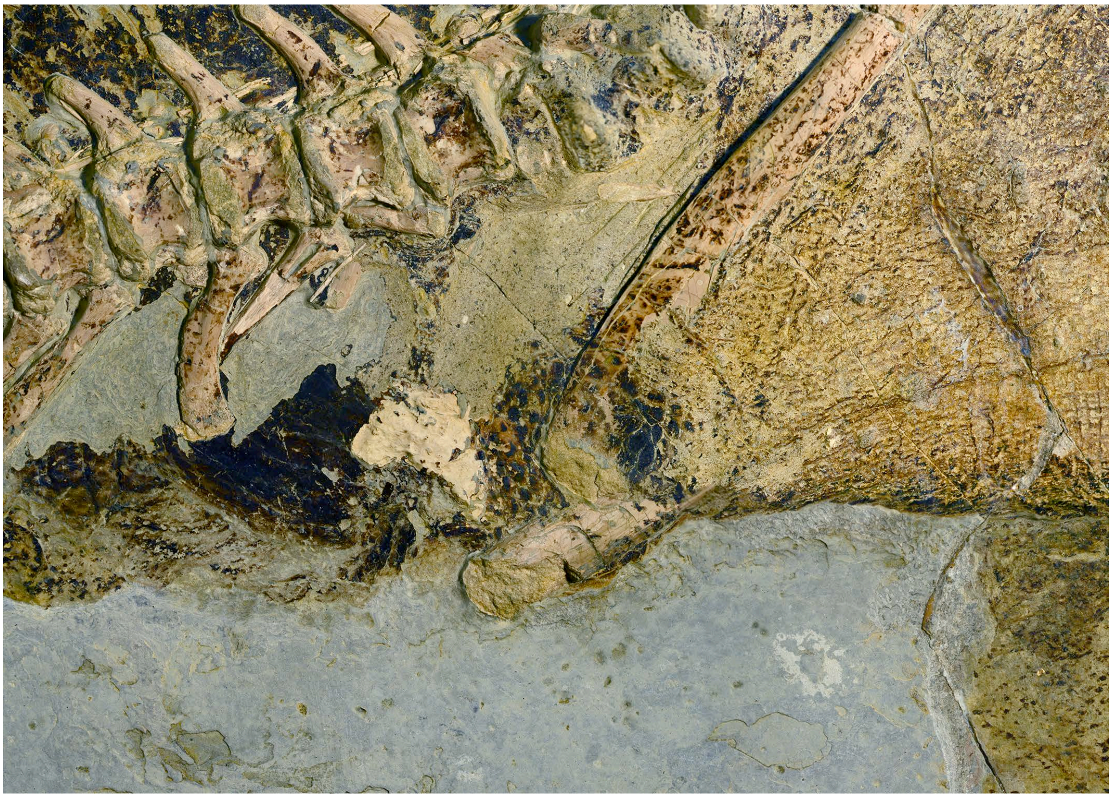

Figure S9. ***Psittacosaurus sp.*** **SMF R 4970 Detail of cloacal region and ischial callosity**. The cream-coloured mass exposed underneath the cloacal pigmented region might be a coprolite.

Reconstruction of *Psittacosaurus*, SMF R 4970

In order produce an anatomically accurate 3D reconstruction of SMF R 4970 the anatomy was sketched in a lateral and dorsal view as a reference for the sculpting process. Firstly, all exposed bones, keratinous appendages, and soft tissue profile of SMF R 4970 were measured on location at the Naturmuseum Senckenberg, Frankfurt. These measurements were used to render detailed drawings of the skeleton in dorsal and lateral views (Figure S10). Muscle groups were added later.

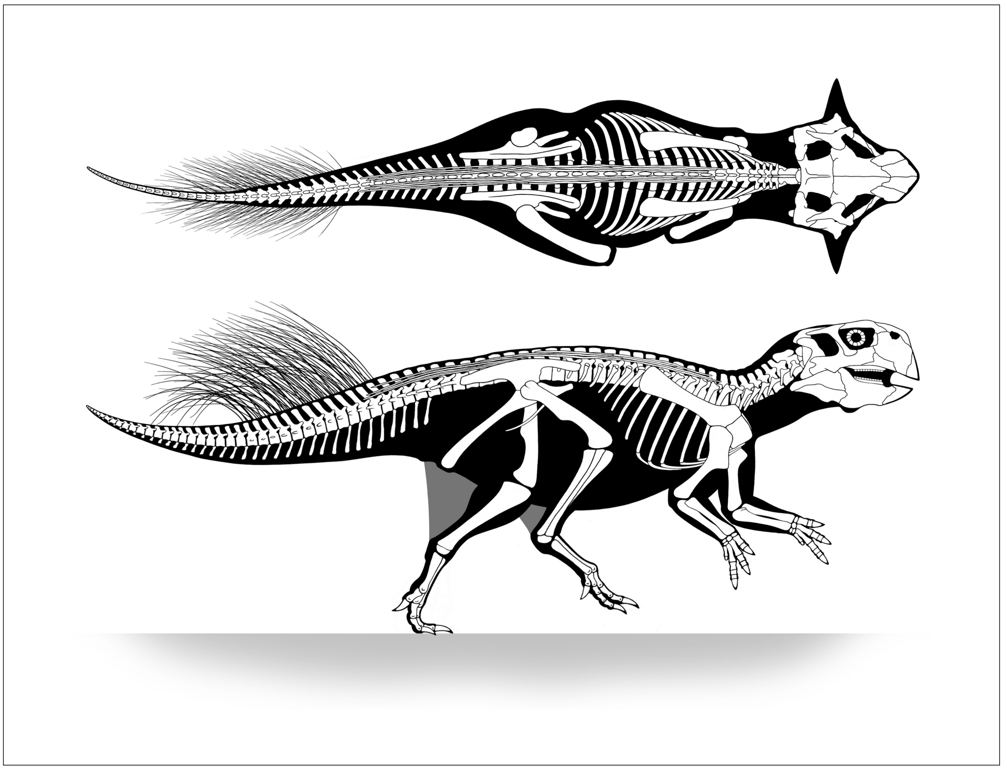

Figure S10. Skeletal reconstruction and body outline of SMF R 4970. Grey shaded areas indicate inferred patagia.

The preliminary drawings revealed a variety of important features that would greatly affect the appearance of the eventual sculpture. Notable skeletal characteristics include robust limbs and ossified tendons supporting the dorsal vertebrae, which implies that the torso was not highly flexible and so adapted for bipedal locomotion, and a proportionally large skull [35-39]. To assist with reconstructing the flattened skull in three dimensions, additional reference was supplied by a 3D print of a skull from *Psittacosaurus lujiatunensis*, and lower jaw (BSPG 2007 I 63), which was CT-scanned, and subsequently retrodeformed and mirrored sagittally to remove preservational artefacts.

Keratinous sheaths

The well preserved presumably keratinous horn on the specimen's left cheek is 4.9 cm long but incomplete. By following the preserved curvature of the horn's margins an approximate total length of 6 cm was calculated, increasing the lateral width of the skull considerably. The bristles on the tail[40], approximately 100 in total, vary in length and taper to points at their distal ends,

but all have a similar (2 mm) diameter where they are overlaid by tail skin. The bristles grow from the tail to create a brush with clear anterior and posterior edges, but the latter is a little obscured by the posterior and ventrally curved bristles. No keratin residues (e.g. melanin, or calcium phosphate salts embedded in the sheath) can be observed on the pes or manus, so the extent of the claw sheaths was made approximately 20% longer than the well-preserved unguals, although this could have been different.

Volume rendering

Soft tissues were added to the preliminary drawings to reveal the external form of the dinosaur in life. Much of the respiratory and digestive systems are contained within the ribcage but considerable volume was added to the abdomen to accommodate a herbivorous stomach and intestines. Volume was also added to the neck to accommodate the oesophagus and trachea. A bulge of tissue was also added to the cloacae, posterior to the ischium (indicated by the dense patch of pigment); its anatomy was modelled on extant birds [41]. Information regarding the nervous and circulatory systems is not preserved and, because these soft tissues have little influence on the external form of extant animals, they were amalgamated into the musculature. The degree of adipose tissue deposits are difficult to infer, based on osteology and have thus been assumed to be minimal (See below for some volume inference based on the fossil). However, the sensory organs – ears, eyes and nostrils – would later be made conspicuous during the sculpting process.

Some information regarding the volume around the skeleton is preserved in SMF R 4970. Measurements from the fossil were adhered to when adding the major muscle groups into the preliminary drawing (Figure S9). Notable muscular characteristics include robust hind and forelimbs, a barrel-like torso, and the base of the tail is broad and powerful, again suggesting the capability to walk bipedally. A particularly unique soft tissue feature of SMF R 4970 is a patagium along the back of each hind limb, running from ankle to the tail. The full extent of the patagium is somewhat obscured by matrix and the overlay of abdominal soft tissue. However, by following the curvature of the exposed posterior margin, it was estimated that the patagium would connect to the tail approximately 10cm

behind the illium along the contour where the *M. caudofemoralis* extends to the greater trochanter.

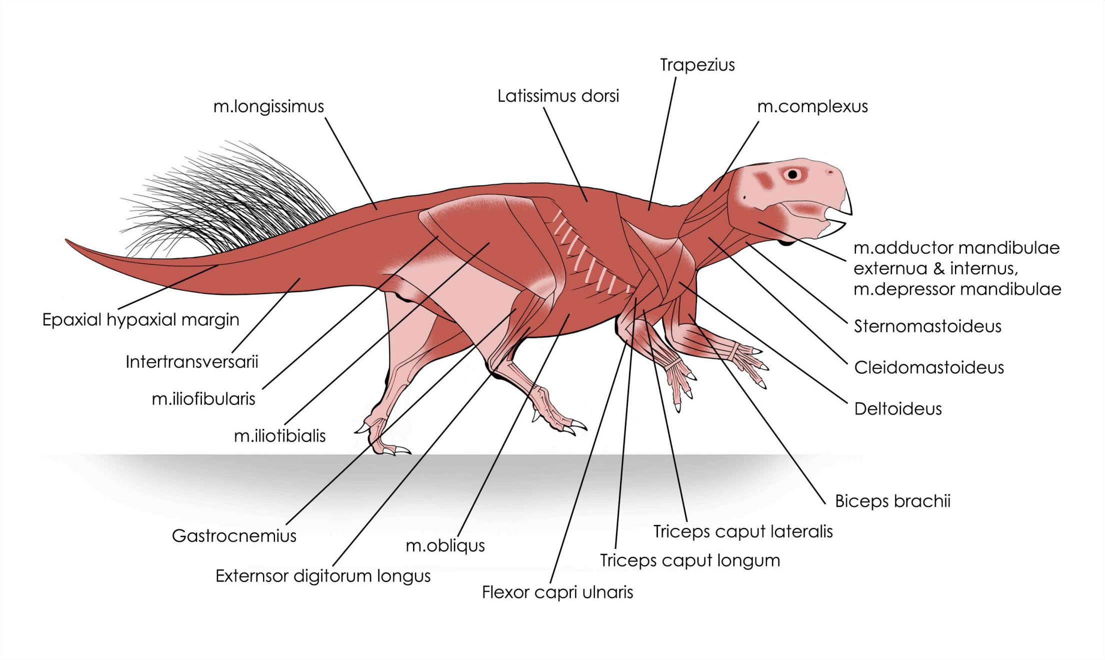

Figure S11. Reconstructed muscle groups on SMF R 4970.

External surface

Integumentary features preserved are scales of varying sizes, resting pads, skin folds, and colour patterns. These were projected onto the model. Unique to this specimen are rounded clusters of highly pigmented scales on the shoulders. Also noteworthy are the well preserved resting pads, some with enlarged scales and some with very small scales. The pads located at the distal end of the ischium (ischial callosity), the knee, ankle, and elbow have large scales. Conversely, the pads on the underside of the pes and manus are small, and the presence of a pad on the dinosaur's left manus suggest *Psittacosaurus* spent at least some of its time walking or standing quadrupedally. However, the alternative option of a bipedal posture was chosen for this active reconstruction, based on the osteological evidence in SMF R 4970, previous studies [42] and the relatively more pigmented chest. Finally, the folds of skin on either side of the neck and on the anterior side of the ankle were recorded in preparation for sculpting.

Modelling process

The completed preliminary drawings were printed on paper at life-size and used as reference when sculpting the 3D reconstruction. All life-size sculptures begin with a supportive armature, and in the case of *Psittacosaurus* it was a welded steel frame connected to the plinth below the right pes and where the left pes would touch the ground (Figure S12). Blocks of polystyrene were cut and fixed to the armature, using expanding foam, and then sculpted into a form 20 mm to 50 mm smaller than that depicted by the preliminary drawings, as if the *Psittacosaurus* had been deflated. Flexible wire was inserted into the polystyrene to create an armature of the forelimbs before wire mesh was tightly wrapped round the whole structure (Figure S13).

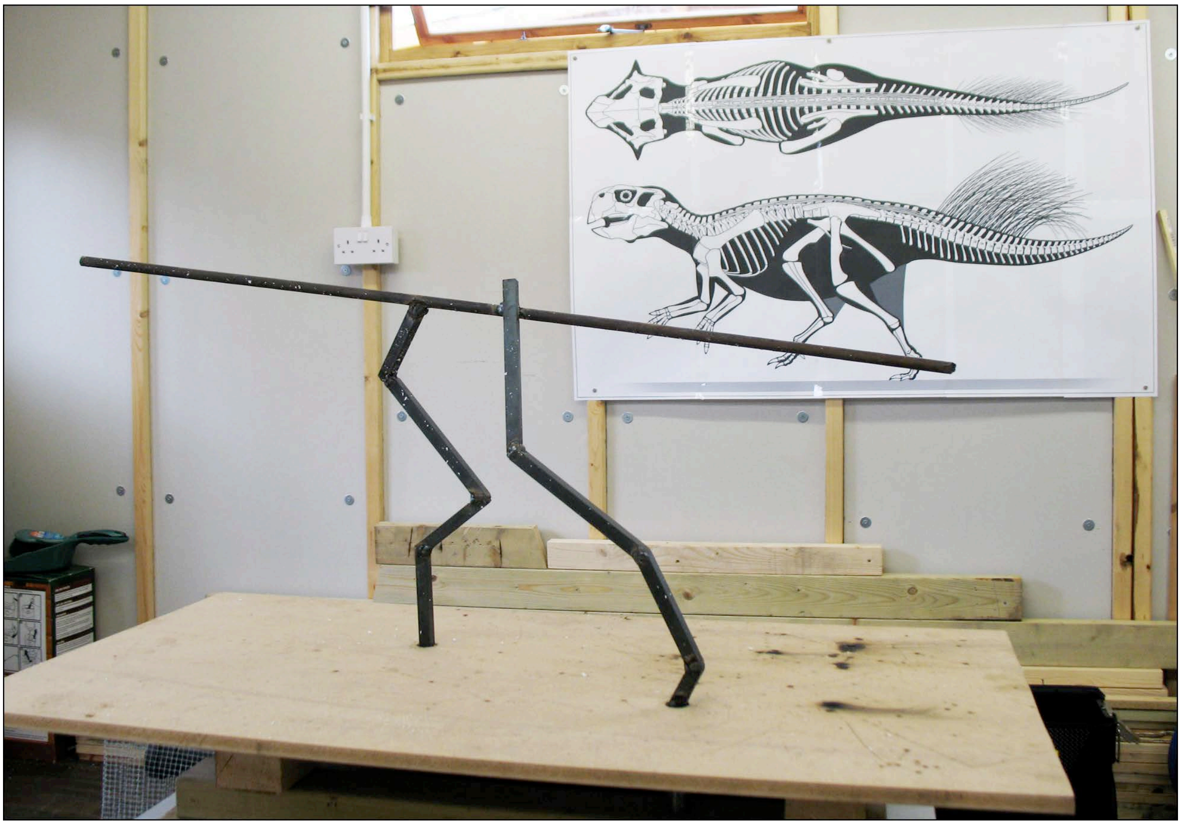

Fig S12. The welded steel frame that formed the basic armature of the model.

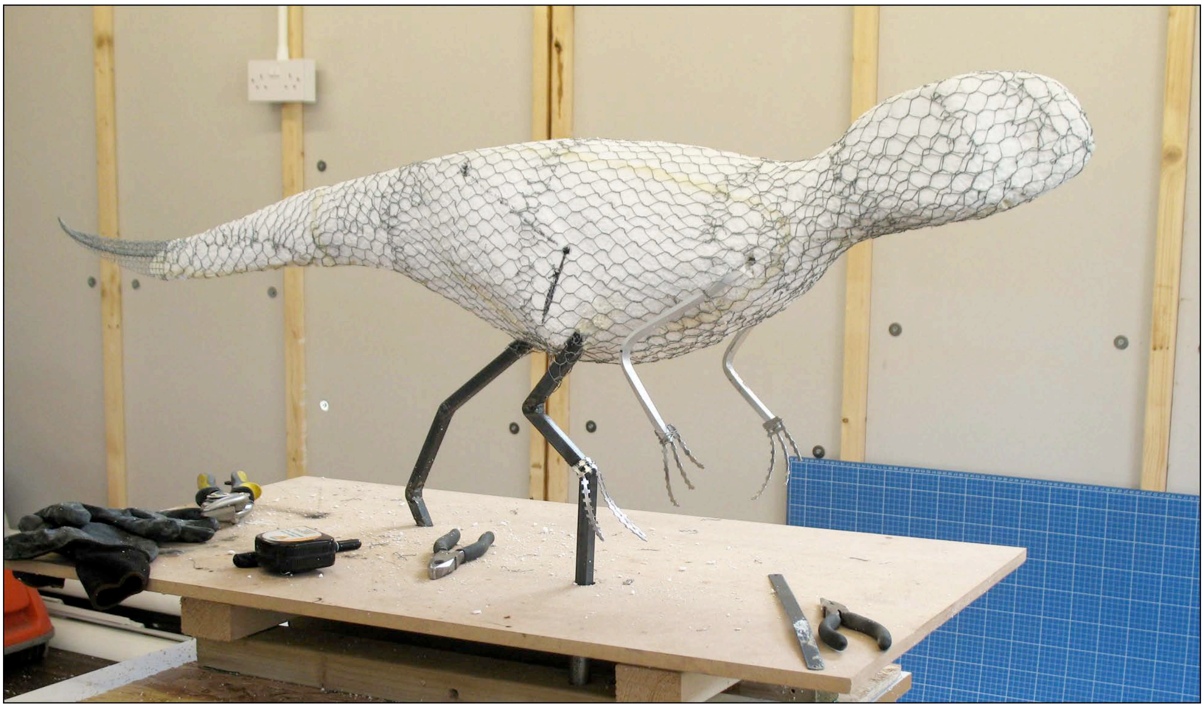

Figure S13. Added polystyrene and wire mesh provide the bulk onto which clay is sculpted.

The clay was added to the structure in 20 mm to 50 mm thicknesses, then adjusted until the proportions precisely matched the dimensions specified by the preliminary drawings. Next, the sensory organs were added. The orbit of *Psittacosaurus sp.* is extremely large but with the inclusion of soft tissue – muscles and eyelids - reduced the eyeball to approximately 28 mm in diameter. The eyeball was sculpted in the centre of the orbit. However, the nostrils were located at the anterior edge of the external naris [43]. The bird-like external ear drum was positioned below the otic notch, between the back of the quadratojugal and the *M. depressor* (Figure S11, S14).

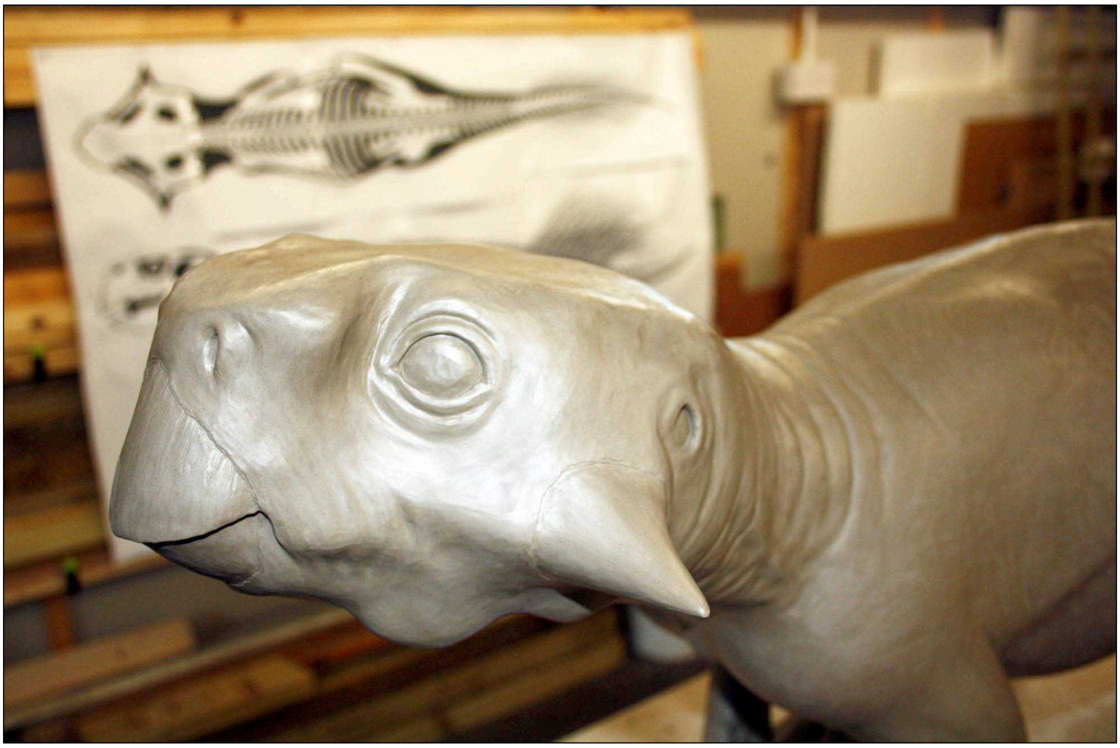

Figure S14. The partially completed head, sculpted in clay.

Based on the integumental information extracted from SMF R 4970 the scales, skin folds, and resting pads were added to the clay surface. Compression wrinkles not present in the fossil were added to the right side of the torso, where the skin is compressed between the limbs during a stride, and to the dorsal side of the neck. Little soft tissue information has been preserved around the mouth of SMF R 4970, but there is some evidence of cheeks due to the presence of highly pigmented integument extending across the lower jaw across to the lateral face (Figure S14). A subtle margin between the hypaxial and epaxial muscle groups was also added to the centre portion of the tail. The completed sculpture was then moulded using silicone rubber and acrylic resin (Figure S15).

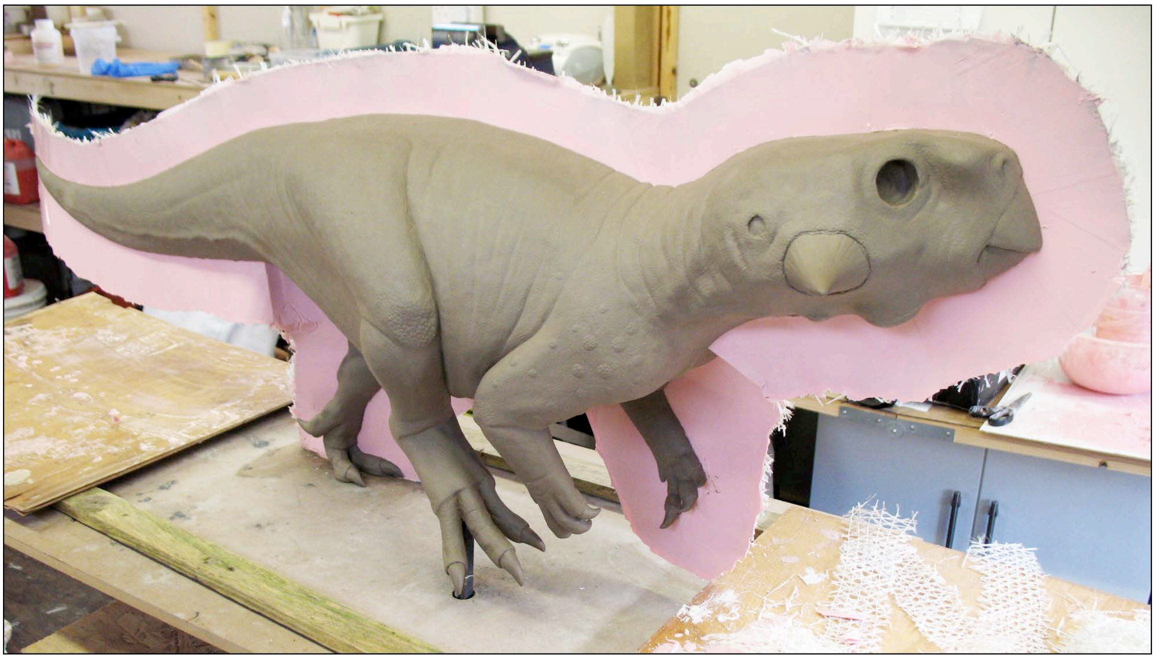

Figure S15. Making the silicone and resin mould from the clay model.

Two fibreglass and resin casts were taken from the mould and repaired where necessary (the section of armature supporting the right pes was removed). One was painted with matt grey paint and did not have bristles attached. This served as the model for analysis of countershading and illumination environment (see below). The second, into which glass eyes were inserted, was used for the colour reconstruction. Choosing pupil shape necessitated considerations of the likely ecology and habitat of *P. lujiatunensis* [44]. A bird-like eye with a round pupil was chosen because a vertical or horizontal slit eye would be suggestive of a predatory and/or nocturnal animal. A horizontal rectangular pupil was also rejected because the Lower Cretaceous environment in northeastern China was not open grassland equivalent. The colour patterns were meticulously applied to the cast using a combination of sponges, brushes, and airbrush, to achieve the required effects. Every square centimetre of the sculpture's surface was carefully considered and the patterns preserved in SMF R 4970 were applied to the precise corresponding locations; subsequently many scales had to be individually half or quarter painted with dark pigment. Finally, the replica bristles were fixed into the tail and bent into curves and positions that mimic those preserved in the SMF R 4970 fossil (Figure S16).

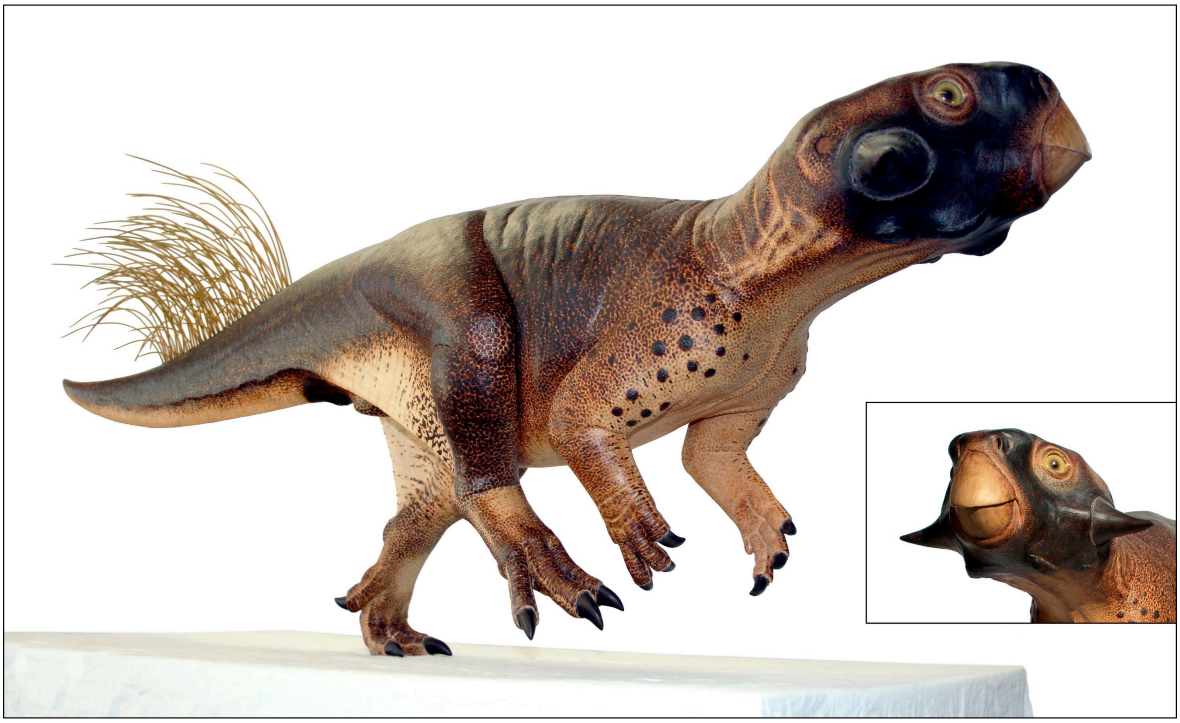

Figure S16. The painted model with tail quills and glass eyes added.

Digital model

In order to provide a digital model of the physical reconstruction in 3D PDF format, a photogrammetry approach was applied. The digitisation was based on 48 photographs from different angles taken with a Nikon D800 digital SLR camera with a 60 mm Nikkor macro lens (Nikon Corporation, Tokyo, Japan). Creation of the digital model was performed in Agisoft Photoscan Standard (www.agisoft.ru). The final digital model consists of 720,000 elements and was exported as an .OBJ file (including texture information). The model was subsequently converted into .U3D format using Adobe 3D Reviewer (Adobe Systems Inc.) to assemble the final 3D PDF files (see supplementary information file Document S1, S2)

Predicting countershading

Following Allen et al. [45], the uniform grey model was photographed under different natural lighting conditions in order to determine the pattern of illumination and cast shadows. The photographs were taken at the University of

Bristol Botanic Gardens (latitude 51.47711 longitude -2.62599) in an area devoted to relict plants typical of the early Cretaceous, including monkey puzzle (*Araucarites*), horsetails (*Equisetites*) and cycads. Photographs were taken near midday (± 2 h), variously in the open and under vegetation, on sunny (<10% cloud cover) and overcast (complete cloud cover) days. In terms of the cast shadows, these variants reduce to two illumination conditions, direct vs diffuse, according to whether the majority of the light is coming directly from the sun's disk or from the sky [45-48]. In all cases the model was oriented with the head and long axis of the body pointing towards the sun. In diffuse illumination orientation has little effect on the optimal countershading but, under direct sun, only facing towards or away from the sun favours symmetrical coloration with respect to an animal's mid-line [48]. As we know of no vertebrates with asymmetric countershading, we felt the orientation constraint reasonable [45].

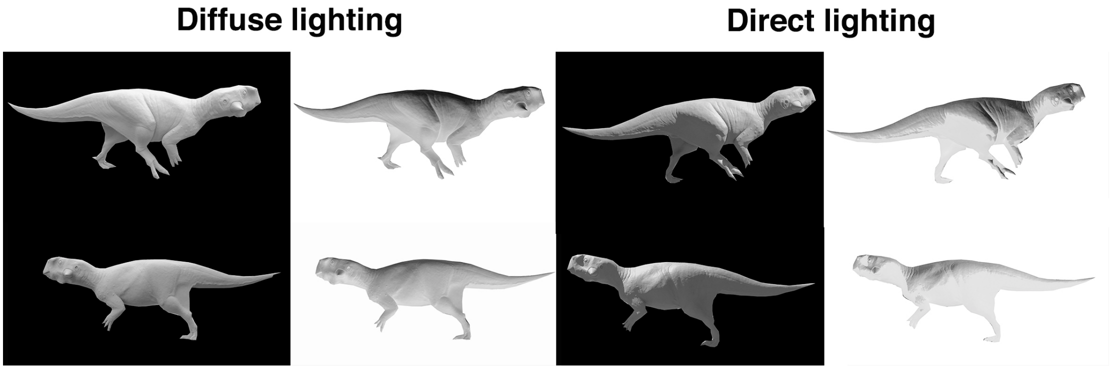

Figure S17. Model photographed in natural light conditions. The images are greyscales, masked and then colours are inverted. Bristles not attached to this model.

Photographs were taken with a Nikon D7100 digital SLR camera with a 28-250 mm Nikkor lens (Nikon Corporation, Tokyo, Japan), set at a focal length of 35 mm (i.e. approximately 1:1 reproduction) on aperture priority at f8 and ISO 200, using automatic focus and metering on the middle of the model's flank. Images were saved in RAW format (Nikon NEF) and subsequently converted to 8-bit TIFF using DCRAW v.9.06 [49]. A colour standard (X-Rite Color Passport; X-Rite Inc. Grand Rapids, MI, USA) was placed next to, and in the same plane as, the

model in all photographs. The 24 colour squares were used to linearize the pixel-value-intensity relationship [50, 51] and, using a quadratic polynomial, map the pixel values to apparent reflectance. By the latter we mean that the reflectance of the brightest part of the model's body was anchored to its real value based on the linearization, then all other intensities (generated by differential illumination) expressed as a proportion of that. Having reconstructed the pattern of illumination, the negative was taken as a predictor of the optimal countershading for self-shadow concealment [47, 48, 52]: the pattern of integument shading that would exactly counterbalance the pattern of illumination. All image manipulation was carried out in Matlab R2013 (The Mathworks Inc., Natick MA, USA)

Supplemental references

1. Sereno, P.C. (2010). Taxonomy, cranial morphology, and relationships of parrot-beaked dinosaurs (Ceratopsia: Psittacosaurus). In New perspectives on horned dinosaurs: The Royal Tyrrell Museum ceratopsian symposium. (Indiana University Press Bloomington), pp. 21-58.
2. Hedrick, B.P., and Dodson, P. (2013). Lujiatun Psittacosaurids: Understanding Individual and Taphonomic Variation Using 3D Geometric Morphometrics. PLoS ONE *8*, e69265.
3. Crabb, P. (2001). The use of polarised light in photography of macrofossils. Palaeontology *44*, 659-664.
4. Kaye, T.G., Falk, A.R., Pittman, M., Sereno, P.C., Martin, L.D., Burnham, D.A., Gong, E., Xu, X., and Wang, Y. (2015). Laser-Stimulated Fluorescence in Paleontology. PloS one *10*, e0125923.
5. Pautard, F. (1964). Calcification of keratin. In Progress in the biological sciences in relation to dermatology, Volume 2. (Cambridge University Press London), p. 227.
6. Foth, C., Tischlinger, H., and Rauhut, O.W. (2014). New specimen of Archaeopteryx provides insights into the evolution of pennaceous feathers. Nature *511*, 79-82.
7. Benton, M.J., Zhonghe, Z., Orr, P.J., Fucheng, Z., and Kearns, S.L. (2008). The remarkable fossils from the Early Cretaceous Jehol Biota of China and how they have changed our knowledge of Mesozoic life: Presidential Address, delivered 2nd May 2008. Proceedings of the Geologists' Association *119*, 209-228.
8. Vinther, J., Briggs, D.E.G., Prum, R.O., and Saranathan, V. (2008). The colour of fossil feathers. Biol Letters *4*, 522-525.
9. Colleary, C., Dolocan, A., Gardner, J., Singh, S., Wuttke, M., Rabenstein, R., Habersetzer, J., Schaal, S., Feseha, M., Clemens, M., et al. (2015). Chemical, experimental, and morphological evidence for diagenetically altered melanin in exceptionally preserved fossils. Proceedings of the National Academy of Sciences.

10. Li, Q., Gao, K.-Q., Meng, Q., Clarke, J.A., Shawkey, M.D., D'Alba, L., Pei, R., Ellison, M., Norell, M.A., and Vinther, J. (2012). Reconstruction of Microraptor and the evolution of iridescent plumage. Science *335*, 1215-1219.
11. Li, Q., Gao, K.-Q., Vinther, J., Shawkey, M.D., Clarke, J.A., D´Alba, L., Meng, Q., Briggs, D.E.G., and Prum, R.O. (2010). Plumage color patterns of an extinct dinosaur. Science *327*, 1369-1372.
12. Zhang, F., Kearns, S.L., Orr, P.J., Benton, M.J., Zhou, Z., Johnson, D., Xu, X., and Wang, X. (2010). Fossilized melanosomes and the colour of Cretaceous dinosaurs and birds. Nature *463*, 1075-1078.
13. Edwards, N., Barden, H., Van Dongen, B., Manning, P., Larson, P., Bergmann, U., Sellers, W., and Wogelius, R. (2011). Infrared mapping resolves soft tissue preservation in 50 million year-old reptile skin. Proceedings of the Royal Society of London B: Biological Sciences *278*, 3209-3218.
14. Schweitzer, M., Watt, J., Avci, R., Knapp, L., Chiappe, L., Norell, M., and Marshall, M. (1999). Beta-keratin specific immunological reactivity in feather-like structures of the Cretaceous alvarezsaurid, *Shuvuuia deserti*. Journal of Experimental Zoology, 146-157.
15. Lingham-Soliar, T., and Plodowski, G. (2010). The integument of *Psittacosaurus* from Liaoning Province, China: taphonomy, epidermal patterns and color of a ceratopsian dinosaur. Naturwissenschaften *97*, 479-486.
16. Wang, S.-Y., Cappellini, E., and Zhang, H.-Y. (2012). Why collagens best survived in fossils? Clues from amino acid thermal stability. Biochemical and Biophysical Research Communications *422*, 5-7.
17. Nance, J.R., Armstrong, J.T., Cody, G.D., Fogel, M.L., and Hazen, R.M. (2015). Preserved macroscopic polymeric sheets of shell-binding protein in the Middle Miocene (8 to 18 Ma) gastropod Ecphora. Geochemical Perspectives Letters *1*, 1-9.
18. Penkman, K., Kaufman, D., Maddy, D., and Collins, M. (2008). Closed-system behaviour of the intra-crystalline fraction of amino acids in mollusc shells. Quaternary Geochronology *3*, 2-25.
19. Vinther, J., Briggs, D.E.G., Clarke, J., Mayr, G., and Prum, R.O. (2010). Structural coloration in a fossil feather. Biol Letters *6*, 128-131.
20. Carney, R.M., Vinther, J., Shawkey, M.D., D'Alba, L., and Ackermann, J. (2012). New evidence on the colour and nature of the isolated *Archaeopteryx* feather. Nature Communications *3*, 637.
21. Vitek, N.S., Vinther, J., Schiffbauer, J.D., Briggs, D.E.G., and Prum, R.O. (2013). Exceptional three-dimensional preservation and coloration of an originally iridescent fossil feather from the Middle Eocene Messel Oil Shale. Palaeontologische Zeitschrift *87*, 493-503.
22. Smith, N.A., Chiappe, L.M., Clarke, J.A., Edwards, S.V., Nesbitt, S.J., Norell, M.A., Stidham, T.A., Turner, A., van Tuinen, M., and Vinther, J. (2015). Rhetoric vs. reality: A commentary on "Bird Origins Anew" by A. Feduccia. The Auk *132*, 467-480.
23. Gupta, N.S., Cody, G.D., Tetlie, O.E., Briggs, D.E., and Summons, R.E. (2009). Rapid incorporation of lipids into macromolecules during experimental

decay of invertebrates: Initiation of geopolymer formation. Organic Geochemistry *40*, 589-594.
24. Field, D.J., D'Alba, L., Vinther, J., Webb, S.M., Gearty, W., and Shawkey, M.D. (2013). Melanin concentration gradients in modern and fossil feathers. Plos One *8*.
25. Vinther, J. (2015). A guide to the field of palaeo colour: Melanin and other pigments can fossilise: Reconstructing colour patterns from ancient organisms can give new insights to ecology and behaviour. Bioessays *37*, 643-656.
26. Li, Q., Clarke, J.A., Gao, K.-Q., Zhou, C.-F., Meng, Q., Li, D., D'Alba, L., and Shawkey, M.D. (2014). Melanosome evolution indicates a key physiological shift within feathered dinosaurs. Nature *507*, 350-353.
27. Schweitzer, M.H., Lindgren, J., and Moyer, A.E. (2015). Melanosomes and ancient coloration re - examined: A response to Vinther 2015 (DOI 10.1002/bies. 201500018). BioEssays.
28. Lindgren, J., Sjövall, P., Carney, R.M., Cincotta, A., Uvdal, P., Hutcheson, S.W., Gustafsson, O., Lefèvre, U., Escuillié, F., and Heimdal, J. (2015). Molecular composition and ultrastructure of Jurassic paravian feathers. Scientific reports *5*.
29. Chen, P.-j., Dong, Z.-m., and Zhen, S.-n. (1998). An exceptionally well-preserved theropod dinosaur from the Yixian Formation of China. Nature *391*, 147-152.
30. Briggs, D.E.G., and Williams, S.H. (1981). The restoration of flattened fossils. Lethaia *14*, 157-164.
31. Bonser, R.H.C. (1995). Melanin and the abrasion resistance of feathers. Condor *97*, 590-591.
32. Bonser, R.H.C., and Witter, M.S. (1993). Indentation hardness of the bill keratin of the European Starling. Condor *95*, 736-738.
33. Mackintosh, J.A. (2001). The antimicrobial properties of melanocytes, melanosomes and melanin and the evolution of black skin. Journal of Theoretical Biology *211*, 101-113.
34. Schiel, G., Corsaro, C., Scalia, M., Sciuto, S., and Geremia, E. (1987). Relationship between melanin content and superoxide dismutase (SOD) activity in the liver of various species of animals. Cell Biochemistry and Function *5*, 123-128.
35. Organ, C.L. (2006). Thoracic epaxial muscles in living archosaurs and ornithopod dinosaurs. The Anatomical Record Part A: Discoveries in Molecular, Cellular, and Evolutionary Biology *288*, 782-793.
36. Senter, P. (2007). Analysis of forelimb function in basal ceratopsians. Journal of Zoology *273*, 305-314.
37. Sereno, P.C., Xijin, Z., Brown, L., and Lin, T. (2007). New psittacosaurid highlights skull enlargement in horned dinosaurs. Acta Palaeontol Pol *52*, 275.
38. Kilbourne, B.M., and Makovicky, P.J. (2010). Limb bone allometry during postnatal ontogeny in non - avian dinosaurs. Journal of Anatomy *217*, 135-152.
39. Zhou, C.F., Gao, K.Q., and Fox, R.C. (2010). Morphology and Histology of Lattice - like Ossified Epaxial Tendons in Psittacosaurus (Dinosauria: Ceratopsia). Acta Geologica Sinica (English edition) *84*, 463-471.

40. Mayr, G., Peters, D.S., Plodowski, G., and Vogel, O. (2002). Bristle-like integumentary structures at the tail of the horned dinosaur Psiffacosaurus. Naturwissenschaften *89*, 361-365.
41. Van Grouw, K. (2013). The Unfeathered Bird, (CSIRO).
42. Zhao, Q., Benton, M.J., Sullivan, C., Sander, P.M., and Xu, X. (2013). Histology and postural change during the growth of the ceratopsian dinosaur Psittacosaurus lujiatunensis. Nature communications *4*.
43. Witmer, L.M. (2001). Nostril position in dinosaurs and other vertebrates and its significance for nasal function. Science *293*, 850-853.
44. Banks, M.S., Sprague, W.W., Schmoll, J., Parnell, J.A.Q., and Love, G.D. (2015). Why do animal eyes have pupils of different shapes? Science Advances *1*.
45. Allen, W.L., Baddeley, R., Cuthill, I.C., and Scott-Samuel, N.E. (2012). A quantitative test of the predicted relationship between countershading and lighting environment. American Naturalist *180*, 762-776.
46. Endler, J.A. (1993). Some general comments on the evolution and design of animal communication systems. Philosophical Transactions of the Royal Society of London, Series B *340*, 215-225.
47. Penacchio, O., Lovell, P.G., Cuthill, I.C., Ruxton, G.D., and Harris, J.M. (2015). Three dimensional camouflage: exploiting photons to conceal form. American Naturalist *186*, 553-563.
48. Penacchio, O., Ruxton, G.D., Lovell, P.G., Cuthill, I.C., and Harris, J.M. (2015). Orientation to the sun by animals and its interaction with crypsis. Functional Ecology *29*, 1165-1177.
49. Coffin, D. Decoding raw digital photos. 2008. URL http://www. cybercom. net/~ dcoffin/dcraw *60*.
50. Westland, S., and Ripamonti, C. (2004). Computational Colour Science using MATLAB, (Chichester, West Sussex: John Wiley & Sons Ltd).
51. Stevens, M., Parraga, C.A., Cuthill, I.C., Partridge, J.C., and Troscianko, T.S. (2007). Using digital photography to study animal coloration. Biological Journal Of The Linnean Society *90*, 211-237.
52. Allen, W.L., Baddeley, R., Cuthill, I.C., and Scott-Samuel, N.E. (2012). A quantitative test of the predicted relationship between countershading and lighting environment. The American Naturalist *180*, 762-776.
